# Measurement of *k*_T_ splitting scales in *W*→*ℓν* events at $\sqrt{s} = 7\ \mathrm{TeV}$ with the ATLAS detector

**DOI:** 10.1140/epjc/s10052-013-2432-8

**Published:** 2013-05-15

**Authors:** The ATLAS Collaboration, G. Aad, T. Abajyan, B. Abbott, J. Abdallah, S. Abdel Khalek, A. A. Abdelalim, O. Abdinov, R. Aben, B. Abi, M. Abolins, O. S. AbouZeid, H. Abramowicz, H. Abreu, B. S. Acharya, L. Adamczyk, D. L. Adams, T. N. Addy, J. Adelman, S. Adomeit, P. Adragna, T. Adye, S. Aefsky, J. A. Aguilar-Saavedra, M. Agustoni, S. P. Ahlen, F. Ahles, A. Ahmad, M. Ahsan, G. Aielli, T. P. A. Åkesson, G. Akimoto, A. V. Akimov, M. A. Alam, J. Albert, S. Albrand, M. Aleksa, I. N. Aleksandrov, F. Alessandria, C. Alexa, G. Alexander, G. Alexandre, T. Alexopoulos, M. Alhroob, M. Aliev, G. Alimonti, J. Alison, B. M. M. Allbrooke, L. J. Allison, P. P. Allport, S. E. Allwood-Spiers, J. Almond, A. Aloisio, R. Alon, A. Alonso, F. Alonso, A. Altheimer, B. Alvarez Gonzalez, M. G. Alviggi, K. Amako, C. Amelung, V. V. Ammosov, S. P. Amor Dos Santos, A. Amorim, S. Amoroso, N. Amram, C. Anastopoulos, L. S. Ancu, N. Andari, T. Andeen, C. F. Anders, G. Anders, K. J. Anderson, A. Andreazza, V. Andrei, X. S. Anduaga, S. Angelidakis, P. Anger, A. Angerami, F. Anghinolfi, A. Anisenkov, N. Anjos, A. Annovi, A. Antonaki, M. Antonelli, A. Antonov, J. Antos, F. Anulli, M. Aoki, L. Aperio Bella, R. Apolle, G. Arabidze, I. Aracena, Y. Arai, A. T. H. Arce, S. Arfaoui, J-F. Arguin, S. Argyropoulos, E. Arik, M. Arik, A. J. Armbruster, O. Arnaez, V. Arnal, A. Artamonov, G. Artoni, D. Arutinov, S. Asai, S. Ask, B. Åsman, L. Asquith, K. Assamagan, R. Astalos, A. Astbury, M. Atkinson, B. Auerbach, E. Auge, K. Augsten, M. Aurousseau, G. Avolio, D. Axen, G. Azuelos, Y. Azuma, M. A. Baak, G. Baccaglioni, C. Bacci, A. M. Bach, H. Bachacou, K. Bachas, M. Backes, M. Backhaus, J. Backus Mayes, E. Badescu, P. Bagnaia, Y. Bai, D. C. Bailey, T. Bain, J. T. Baines, O. K. Baker, S. Baker, P. Balek, F. Balli, E. Banas, P. Banerjee, Sw. Banerjee, D. Banfi, A. Bangert, V. Bansal, H. S. Bansil, L. Barak, S. P. Baranov, T. Barber, E. L. Barberio, D. Barberis, M. Barbero, D. Y. Bardin, T. Barillari, M. Barisonzi, T. Barklow, N. Barlow, B. M. Barnett, R. M. Barnett, A. Baroncelli, G. Barone, A. J. Barr, F. Barreiro, J. Barreiro Guimarães da Costa, R. Bartoldus, A. E. Barton, V. Bartsch, A. Basye, R. L. Bates, L. Batkova, J. R. Batley, A. Battaglia, M. Battistin, F. Bauer, H. S. Bawa, S. Beale, T. Beau, P. H. Beauchemin, R. Beccherle, P. Bechtle, H. P. Beck, K. Becker, S. Becker, M. Beckingham, K. H. Becks, A. J. Beddall, A. Beddall, S. Bedikian, V. A. Bednyakov, C. P. Bee, L. J. Beemster, T. A. Beermann, M. Begel, S. Behar Harpaz, C. Belanger-Champagne, P. J. Bell, W. H. Bell, G. Bella, L. Bellagamba, M. Bellomo, A. Belloni, O. Beloborodova, K. Belotskiy, O. Beltramello, O. Benary, D. Benchekroun, K. Bendtz, N. Benekos, Y. Benhammou, E. Benhar Noccioli, J. A. Benitez Garcia, D. P. Benjamin, M. Benoit, J. R. Bensinger, K. Benslama, S. Bentvelsen, D. Berge, E. Bergeaas Kuutmann, N. Berger, F. Berghaus, E. Berglund, J. Beringer, P. Bernat, R. Bernhard, C. Bernius, F. U. Bernlochner, T. Berry, C. Bertella, A. Bertin, F. Bertolucci, M. I. Besana, G. J. Besjes, N. Besson, S. Bethke, W. Bhimji, R. M. Bianchi, L. Bianchini, M. Bianco, O. Biebel, S. P. Bieniek, K. Bierwagen, J. Biesiada, M. Biglietti, H. Bilokon, M. Bindi, S. Binet, A. Bingul, C. Bini, C. Biscarat, B. Bittner, C. W. Black, J. E. Black, K. M. Black, R. E. Blair, J.-B. Blanchard, T. Blazek, I. Bloch, C. Blocker, J. Blocki, W. Blum, U. Blumenschein, G. J. Bobbink, V. S. Bobrovnikov, S. S. Bocchetta, A. Bocci, C. R. Boddy, M. Boehler, J. Boek, T. T. Boek, N. Boelaert, J. A. Bogaerts, A. Bogdanchikov, A. Bogouch, C. Bohm, J. Bohm, V. Boisvert, T. Bold, V. Boldea, N. M. Bolnet, M. Bomben, M. Bona, M. Boonekamp, S. Bordoni, C. Borer, A. Borisov, G. Borissov, I. Borjanovic, M. Borri, S. Borroni, J. Bortfeldt, V. Bortolotto, K. Bos, D. Boscherini, M. Bosman, H. Boterenbrood, J. Bouchami, J. Boudreau, E. V. Bouhova-Thacker, D. Boumediene, C. Bourdarios, N. Bousson, S. Boutouil, A. Boveia, J. Boyd, I. R. Boyko, I. Bozovic-Jelisavcic, J. Bracinik, P. Branchini, A. Brandt, G. Brandt, O. Brandt, U. Bratzler, B. Brau, J. E. Brau, H. M. Braun, S. F. Brazzale, B. Brelier, J. Bremer, K. Brendlinger, R. Brenner, S. Bressler, T. M. Bristow, D. Britton, F. M. Brochu, I. Brock, R. Brock, F. Broggi, C. Bromberg, J. Bronner, G. Brooijmans, T. Brooks, W. K. Brooks, G. Brown, P. A. Bruckman de Renstrom, D. Bruncko, R. Bruneliere, S. Brunet, A. Bruni, G. Bruni, M. Bruschi, L. Bryngemark, T. Buanes, Q. Buat, F. Bucci, J. Buchanan, P. Buchholz, R. M. Buckingham, A. G. Buckley, S. I. Buda, I. A. Budagov, B. Budick, L. Bugge, O. Bulekov, A. C. Bundock, M. Bunse, T. Buran, H. Burckhart, S. Burdin, T. Burgess, S. Burke, E. Busato, V. Büscher, P. Bussey, C. P. Buszello, B. Butler, J. M. Butler, C. M. Buttar, J. M. Butterworth, W. Buttinger, M. Byszewski, S. Cabrera Urbán, D. Caforio, O. Cakir, P. Calafiura, G. Calderini, P. Calfayan, R. Calkins, L. P. Caloba, R. Caloi, D. Calvet, S. Calvet, R. Camacho Toro, P. Camarri, D. Cameron, L. M. Caminada, R. Caminal Armadans, S. Campana, M. Campanelli, V. Canale, F. Canelli, A. Canepa, J. Cantero, R. Cantrill, T. Cao, M. D. M. Capeans Garrido, I. Caprini, M. Caprini, D. Capriotti, M. Capua, R. Caputo, R. Cardarelli, T. Carli, G. Carlino, L. Carminati, S. Caron, E. Carquin, G. D. Carrillo-Montoya, A. A. Carter, J. R. Carter, J. Carvalho, D. Casadei, M. P. Casado, M. Cascella, C. Caso, E. Castaneda-Miranda, V. Castillo Gimenez, N. F. Castro, G. Cataldi, P. Catastini, A. Catinaccio, J. R. Catmore, A. Cattai, G. Cattani, S. Caughron, V. Cavaliere, P. Cavalleri, D. Cavalli, M. Cavalli-Sforza, V. Cavasinni, F. Ceradini, A. S. Cerqueira, A. Cerri, L. Cerrito, F. Cerutti, S. A. Cetin, A. Chafaq, D. Chakraborty, I. Chalupkova, K. Chan, P. Chang, B. Chapleau, J. D. Chapman, J. W. Chapman, D. G. Charlton, V. Chavda, C. A. Chavez Barajas, S. Cheatham, S. Chekanov, S. V. Chekulaev, G. A. Chelkov, M. A. Chelstowska, C. Chen, H. Chen, S. Chen, X. Chen, Y. Chen, Y. Cheng, A. Cheplakov, R. Cherkaoui El Moursli, V. Chernyatin, E. Cheu, S. L. Cheung, L. Chevalier, G. Chiefari, L. Chikovani, J. T. Childers, A. Chilingarov, G. Chiodini, A. S. Chisholm, R. T. Chislett, A. Chitan, M. V. Chizhov, G. Choudalakis, S. Chouridou, B. K. B. Chow, I. A. Christidi, A. Christov, D. Chromek-Burckhart, M. L. Chu, J. Chudoba, G. Ciapetti, A. K. Ciftci, R. Ciftci, D. Cinca, V. Cindro, A. Ciocio, M. Cirilli, P. Cirkovic, Z. H. Citron, M. Citterio, M. Ciubancan, A. Clark, P. J. Clark, R. N. Clarke, W. Cleland, J. C. Clemens, B. Clement, C. Clement, Y. Coadou, M. Cobal, A. Coccaro, J. Cochran, L. Coffey, J. G. Cogan, J. Coggeshall, J. Colas, S. Cole, A. P. Colijn, N. J. Collins, C. Collins-Tooth, J. Collot, T. Colombo, G. Colon, G. Compostella, P. Conde Muiño, E. Coniavitis, M. C. Conidi, S. M. Consonni, V. Consorti, S. Constantinescu, C. Conta, G. Conti, F. Conventi, M. Cooke, B. D. Cooper, A. M. Cooper-Sarkar, N. J. Cooper-Smith, K. Copic, T. Cornelissen, M. Corradi, F. Corriveau, A. Cortes-Gonzalez, G. Cortiana, G. Costa, M. J. Costa, D. Costanzo, D. Côté, G. Cottin, L. Courneyea, G. Cowan, B. E. Cox, K. Cranmer, S. Crépé-Renaudin, F. Crescioli, M. Cristinziani, G. Crosetti, C.-M. Cuciuc, C. Cuenca Almenar, T. Cuhadar Donszelmann, J. Cummings, M. Curatolo, C. J. Curtis, C. Cuthbert, P. Cwetanski, H. Czirr, P. Czodrowski, Z. Czyczula, S. D’Auria, M. D’Onofrio, A. D’Orazio, M. J. Da Cunha Sargedas De Sousa, C. Da Via, W. Dabrowski, A. Dafinca, T. Dai, F. Dallaire, C. Dallapiccola, M. Dam, D. S. Damiani, H. O. Danielsson, V. Dao, G. Darbo, G. L. Darlea, S. Darmora, J. A. Dassoulas, W. Davey, T. Davidek, N. Davidson, R. Davidson, E. Davies, M. Davies, O. Davignon, A. R. Davison, Y. Davygora, E. Dawe, I. Dawson, R. K. Daya-Ishmukhametova, K. De, R. de Asmundis, S. De Castro, S. De Cecco, J. de Graat, N. De Groot, P. de Jong, C. De La Taille, H. De la Torre, F. De Lorenzi, L. De Nooij, D. De Pedis, A. De Salvo, U. De Sanctis, A. De Santo, J. B. De Vivie De Regie, G. De Zorzi, W. J. Dearnaley, R. Debbe, C. Debenedetti, B. Dechenaux, D. V. Dedovich, J. Degenhardt, J. Del Peso, T. Del Prete, T. Delemontex, M. Deliyergiyev, A. Dell’Acqua, L. Dell’Asta, M. Della Pietra, D. della Volpe, M. Delmastro, P. A. Delsart, C. Deluca, S. Demers, M. Demichev, B. Demirkoz, S. P. Denisov, D. Derendarz, J. E. Derkaoui, F. Derue, P. Dervan, K. Desch, P. O. Deviveiros, A. Dewhurst, B. DeWilde, S. Dhaliwal, R. Dhullipudi, A. Di Ciaccio, L. Di Ciaccio, C. Di Donato, A. Di Girolamo, B. Di Girolamo, S. Di Luise, A. Di Mattia, B. Di Micco, R. Di Nardo, A. Di Simone, R. Di Sipio, M. A. Diaz, E. B. Diehl, J. Dietrich, T. A. Dietzsch, S. Diglio, K. Dindar Yagci, J. Dingfelder, F. Dinut, C. Dionisi, P. Dita, S. Dita, F. Dittus, F. Djama, T. Djobava, M. A. B. do Vale, A. Do Valle Wemans, T. K. O. Doan, M. Dobbs, D. Dobos, E. Dobson, J. Dodd, C. Doglioni, T. Doherty, T. Dohmae, Y. Doi, J. Dolejsi, Z. Dolezal, B. A. Dolgoshein, M. Donadelli, J. Donini, J. Dopke, A. Doria, A. Dos Anjos, A. Dotti, M. T. Dova, A. T. Doyle, N. Dressnandt, M. Dris, J. Dubbert, S. Dube, E. Dubreuil, E. Duchovni, G. Duckeck, D. Duda, A. Dudarev, F. Dudziak, I. P. Duerdoth, L. Duflot, M-A. Dufour, L. Duguid, M. Dührssen, M. Dunford, H. Duran Yildiz, M. Düren, R. Duxfield, M. Dwuznik, W. L. Ebenstein, J. Ebke, S. Eckweiler, W. Edson, C. A. Edwards, N. C. Edwards, W. Ehrenfeld, T. Eifert, G. Eigen, K. Einsweiler, E. Eisenhandler, T. Ekelof, M. El Kacimi, M. Ellert, S. Elles, F. Ellinghaus, K. Ellis, N. Ellis, J. Elmsheuser, M. Elsing, D. Emeliyanov, Y. Enari, R. Engelmann, A. Engl, B. Epp, J. Erdmann, A. Ereditato, D. Eriksson, J. Ernst, M. Ernst, J. Ernwein, D. Errede, S. Errede, E. Ertel, M. Escalier, H. Esch, C. Escobar, X. Espinal Curull, B. Esposito, F. Etienne, A. I. Etienvre, E. Etzion, D. Evangelakou, H. Evans, L. Fabbri, C. Fabre, G. Facini, R. M. Fakhrutdinov, S. Falciano, Y. Fang, M. Fanti, A. Farbin, A. Farilla, J. Farley, T. Farooque, S. Farrell, S. M. Farrington, P. Farthouat, F. Fassi, P. Fassnacht, D. Fassouliotis, B. Fatholahzadeh, A. Favareto, L. Fayard, P. Federic, O. L. Fedin, W. Fedorko, M. Fehling-Kaschek, L. Feligioni, C. Feng, E. J. Feng, A. B. Fenyuk, J. Ferencei, W. Fernando, S. Ferrag, J. Ferrando, V. Ferrara, A. Ferrari, P. Ferrari, R. Ferrari, D. E. Ferreira de Lima, A. Ferrer, D. Ferrere, C. Ferretti, A. Ferretto Parodi, M. Fiascaris, F. Fiedler, A. Filipčič, F. Filthaut, M. Fincke-Keeler, M. C. N. Fiolhais, L. Fiorini, A. Firan, J. Fischer, M. J. Fisher, E. A. Fitzgerald, M. Flechl, I. Fleck, P. Fleischmann, S. Fleischmann, G. T. Fletcher, G. Fletcher, T. Flick, A. Floderus, L. R. Flores Castillo, A. C. Florez Bustos, M. J. Flowerdew, T. Fonseca Martin, A. Formica, A. Forti, D. Fortin, D. Fournier, A. J. Fowler, H. Fox, P. Francavilla, M. Franchini, S. Franchino, D. Francis, T. Frank, M. Franklin, S. Franz, M. Fraternali, S. Fratina, S. T. French, C. Friedrich, F. Friedrich, D. Froidevaux, J. A. Frost, C. Fukunaga, E. Fullana Torregrosa, B. G. Fulsom, J. Fuster, C. Gabaldon, O. Gabizon, S. Gadatsch, T. Gadfort, S. Gadomski, G. Gagliardi, P. Gagnon, C. Galea, B. Galhardo, E. J. Gallas, V. Gallo, B. J. Gallop, P. Gallus, K. K. Gan, R. P. Gandrajula, Y. S. Gao, A. Gaponenko, F. M. Garay Walls, F. Garberson, C. García, J. E. García Navarro, M. Garcia-Sciveres, R. W. Gardner, N. Garelli, V. Garonne, C. Gatti, G. Gaudio, B. Gaur, L. Gauthier, P. Gauzzi, I. L. Gavrilenko, C. Gay, G. Gaycken, E. N. Gazis, P. Ge, Z. Gecse, C. N. P. Gee, D. A. A. Geerts, Ch. Geich-Gimbel, K. Gellerstedt, C. Gemme, A. Gemmell, M. H. Genest, S. Gentile, M. George, S. George, D. Gerbaudo, P. Gerlach, A. Gershon, C. Geweniger, H. Ghazlane, N. Ghodbane, B. Giacobbe, S. Giagu, V. Giangiobbe, F. Gianotti, B. Gibbard, A. Gibson, S. M. Gibson, M. Gilchriese, T. P. S. Gillam, D. Gillberg, A. R. Gillman, D. M. Gingrich, J. Ginzburg, N. Giokaris, M. P. Giordani, R. Giordano, F. M. Giorgi, P. Giovannini, P. F. Giraud, D. Giugni, M. Giunta, B. K. Gjelsten, L. K. Gladilin, C. Glasman, J. Glatzer, A. Glazov, G. L. Glonti, J. R. Goddard, J. Godfrey, J. Godlewski, M. Goebel, C. Goeringer, S. Goldfarb, T. Golling, D. Golubkov, A. Gomes, L. S. Gomez Fajardo, R. Gonçalo, J. Goncalves Pinto Firmino Da Costa, L. Gonella, S. González de la Hoz, G. Gonzalez Parra, M. L. Gonzalez Silva, S. Gonzalez-Sevilla, J. J. Goodson, L. Goossens, T. Göpfert, P. A. Gorbounov, H. A. Gordon, I. Gorelov, G. Gorfine, B. Gorini, E. Gorini, A. Gorišek, E. Gornicki, A. T. Goshaw, C. Gössling, M. I. Gostkin, I. Gough Eschrich, M. Gouighri, D. Goujdami, M. P. Goulette, A. G. Goussiou, C. Goy, S. Gozpinar, L. Graber, I. Grabowska-Bold, P. Grafström, K-J. Grahn, E. Gramstad, F. Grancagnolo, S. Grancagnolo, V. Grassi, V. Gratchev, H. M. Gray, J. A. Gray, E. Graziani, O. G. Grebenyuk, T. Greenshaw, Z. D. Greenwood, K. Gregersen, I. M. Gregor, P. Grenier, J. Griffiths, N. Grigalashvili, A. A. Grillo, K. Grimm, S. Grinstein, Ph. Gris, Y. V. Grishkevich, J.-F. Grivaz, J. P. Grohs, A. Grohsjean, E. Gross, J. Grosse-Knetter, J. Groth-Jensen, K. Grybel, D. Guest, O. Gueta, C. Guicheney, E. Guido, T. Guillemin, S. Guindon, U. Gul, J. Gunther, B. Guo, J. Guo, P. Gutierrez, N. Guttman, O. Gutzwiller, C. Guyot, C. Gwenlan, C. B. Gwilliam, A. Haas, S. Haas, C. Haber, H. K. Hadavand, D. R. Hadley, P. Haefner, Z. Hajduk, H. Hakobyan, D. Hall, G. Halladjian, K. Hamacher, P. Hamal, K. Hamano, M. Hamer, A. Hamilton, S. Hamilton, L. Han, K. Hanagaki, K. Hanawa, M. Hance, C. Handel, P. Hanke, J. R. Hansen, J. B. Hansen, J. D. Hansen, P. H. Hansen, P. Hansson, K. Hara, T. Harenberg, S. Harkusha, D. Harper, R. D. Harrington, O. M. Harris, J. Hartert, F. Hartjes, T. Haruyama, A. Harvey, S. Hasegawa, Y. Hasegawa, S. Hassani, S. Haug, M. Hauschild, R. Hauser, M. Havranek, C. M. Hawkes, R. J. Hawkings, A. D. Hawkins, T. Hayakawa, T. Hayashi, D. Hayden, C. P. Hays, H. S. Hayward, S. J. Haywood, S. J. Head, T. Heck, V. Hedberg, L. Heelan, S. Heim, B. Heinemann, S. Heisterkamp, L. Helary, C. Heller, M. Heller, S. Hellman, D. Hellmich, C. Helsens, R. C. W. Henderson, M. Henke, A. Henrichs, A. M. Henriques Correia, S. Henrot-Versille, C. Hensel, C. M. Hernandez, Y. Hernández Jiménez, R. Herrberg, G. Herten, R. Hertenberger, L. Hervas, G. G. Hesketh, N. P. Hessey, R. Hickling, E. Higón-Rodriguez, J. C. Hill, K. H. Hiller, S. Hillert, S. J. Hillier, I. Hinchliffe, E. Hines, M. Hirose, F. Hirsch, D. Hirschbuehl, J. Hobbs, N. Hod, M. C. Hodgkinson, P. Hodgson, A. Hoecker, M. R. Hoeferkamp, J. Hoffman, D. Hoffmann, M. Hohlfeld, S. O. Holmgren, T. Holy, J. L. Holzbauer, T. M. Hong, L. Hooft van Huysduynen, J-Y. Hostachy, S. Hou, A. Hoummada, J. Howard, J. Howarth, M. Hrabovsky, I. Hristova, J. Hrivnac, T. Hryn’ova, P. J. Hsu, S.-C. Hsu, D. Hu, Z. Hubacek, F. Hubaut, F. Huegging, A. Huettmann, T. B. Huffman, E. W. Hughes, G. Hughes, M. Huhtinen, T. A. Hülsing, M. Hurwitz, N. Huseynov, J. Huston, J. Huth, G. Iacobucci, G. Iakovidis, M. Ibbotson, I. Ibragimov, L. Iconomidou-Fayard, J. Idarraga, P. Iengo, O. Igonkina, Y. Ikegami, K. Ikematsu, M. Ikeno, D. Iliadis, N. Ilic, T. Ince, P. Ioannou, M. Iodice, K. Iordanidou, V. Ippolito, A. Irles Quiles, C. Isaksson, M. Ishino, M. Ishitsuka, R. Ishmukhametov, C. Issever, S. Istin, A. V. Ivashin, W. Iwanski, H. Iwasaki, J. M. Izen, V. Izzo, B. Jackson, J. N. Jackson, P. Jackson, M. R. Jaekel, V. Jain, K. Jakobs, S. Jakobsen, T. Jakoubek, J. Jakubek, D. O. Jamin, D. K. Jana, E. Jansen, H. Jansen, J. Janssen, A. Jantsch, M. Janus, R. C. Jared, G. Jarlskog, L. Jeanty, G.-Y. Jeng, I. Jen-La Plante, D. Jennens, P. Jenni, C. Jeske, P. Jež, S. Jézéquel, M. K. Jha, H. Ji, W. Ji, J. Jia, Y. Jiang, M. Jimenez Belenguer, S. Jin, O. Jinnouchi, M. D. Joergensen, D. Joffe, M. Johansen, K. E. Johansson, P. Johansson, S. Johnert, K. A. Johns, K. Jon-And, G. Jones, R. W. L. Jones, T. J. Jones, C. Joram, P. M. Jorge, K. D. Joshi, J. Jovicevic, T. Jovin, X. Ju, C. A. Jung, R. M. Jungst, V. Juranek, P. Jussel, A. Juste Rozas, S. Kabana, M. Kaci, A. Kaczmarska, P. Kadlecik, M. Kado, H. Kagan, M. Kagan, E. Kajomovitz, S. Kalinin, S. Kama, N. Kanaya, M. Kaneda, S. Kaneti, T. Kanno, V. A. Kantserov, J. Kanzaki, B. Kaplan, A. Kapliy, D. Kar, M. Karagounis, K. Karakostas, M. Karnevskiy, V. Kartvelishvili, A. N. Karyukhin, L. Kashif, G. Kasieczka, R. D. Kass, A. Kastanas, Y. Kataoka, J. Katzy, V. Kaushik, K. Kawagoe, T. Kawamoto, G. Kawamura, S. Kazama, V. F. Kazanin, M. Y. Kazarinov, R. Keeler, P. T. Keener, R. Kehoe, M. Keil, J. S. Keller, M. Kenyon, H. Keoshkerian, O. Kepka, N. Kerschen, B. P. Kerševan, S. Kersten, K. Kessoku, J. Keung, F. Khalil-zada, H. Khandanyan, A. Khanov, D. Kharchenko, A. Khodinov, A. Khomich, T. J. Khoo, G. Khoriauli, A. Khoroshilov, V. Khovanskiy, E. Khramov, J. Khubua, H. Kim, S. H. Kim, N. Kimura, O. Kind, B. T. King, M. King, R. S. B. King, J. Kirk, A. E. Kiryunin, T. Kishimoto, D. Kisielewska, T. Kitamura, T. Kittelmann, K. Kiuchi, E. Kladiva, M. Klein, U. Klein, K. Kleinknecht, M. Klemetti, A. Klier, P. Klimek, A. Klimentov, R. Klingenberg, J. A. Klinger, E. B. Klinkby, T. Klioutchnikova, P. F. Klok, S. Klous, E.-E. Kluge, T. Kluge, P. Kluit, S. Kluth, E. Kneringer, E. B. F. G. Knoops, A. Knue, B. R. Ko, T. Kobayashi, M. Kobel, M. Kocian, P. Kodys, S. Koenig, F. Koetsveld, P. Koevesarki, T. Koffas, E. Koffeman, L. A. Kogan, S. Kohlmann, F. Kohn, Z. Kohout, T. Kohriki, T. Koi, H. Kolanoski, V. Kolesnikov, I. Koletsou, J. Koll, A. A. Komar, Y. Komori, T. Kondo, K. Köneke, A. C. König, T. Kono, A. I. Kononov, R. Konoplich, N. Konstantinidis, R. Kopeliansky, S. Koperny, L. Köpke, A. K. Kopp, K. Korcyl, K. Kordas, A. Korn, A. Korol, I. Korolkov, E. V. Korolkova, V. A. Korotkov, O. Kortner, S. Kortner, V. V. Kostyukhin, S. Kotov, V. M. Kotov, A. Kotwal, C. Kourkoumelis, V. Kouskoura, A. Koutsman, R. Kowalewski, T. Z. Kowalski, W. Kozanecki, A. S. Kozhin, V. Kral, V. A. Kramarenko, G. Kramberger, M. W. Krasny, A. Krasznahorkay, J. K. Kraus, A. Kravchenko, S. Kreiss, F. Krejci, J. Kretzschmar, K. Kreutzfeldt, N. Krieger, P. Krieger, K. Kroeninger, H. Kroha, J. Kroll, J. Kroseberg, J. Krstic, U. Kruchonak, H. Krüger, T. Kruker, N. Krumnack, Z. V. Krumshteyn, M. K. Kruse, T. Kubota, S. Kuday, S. Kuehn, A. Kugel, T. Kuhl, V. Kukhtin, Y. Kulchitsky, S. Kuleshov, M. Kuna, J. Kunkle, A. Kupco, H. Kurashige, M. Kurata, Y. A. Kurochkin, V. Kus, E. S. Kuwertz, M. Kuze, J. Kvita, R. Kwee, A. La Rosa, L. La Rotonda, L. Labarga, S. Lablak, C. Lacasta, F. Lacava, J. Lacey, H. Lacker, D. Lacour, V. R. Lacuesta, E. Ladygin, R. Lafaye, B. Laforge, T. Lagouri, S. Lai, E. Laisne, L. Lambourne, C. L. Lampen, W. Lampl, E. Lançon, U. Landgraf, M. P. J. Landon, V. S. Lang, C. Lange, A. J. Lankford, F. Lanni, K. Lantzsch, A. Lanza, S. Laplace, C. Lapoire, J. F. Laporte, T. Lari, A. Larner, M. Lassnig, P. Laurelli, V. Lavorini, W. Lavrijsen, P. Laycock, O. Le Dortz, E. Le Guirriec, E. Le Menedeu, T. LeCompte, F. Ledroit-Guillon, H. Lee, J. S. H. Lee, S. C. Lee, L. Lee, M. Lefebvre, M. Legendre, F. Legger, C. Leggett, M. Lehmacher, G. Lehmann Miotto, A. G. Leister, M. A. L. Leite, R. Leitner, D. Lellouch, B. Lemmer, V. Lendermann, K. J. C. Leney, T. Lenz, G. Lenzen, B. Lenzi, K. Leonhardt, S. Leontsinis, F. Lepold, C. Leroy, J-R. Lessard, C. G. Lester, C. M. Lester, J. Levêque, D. Levin, L. J. Levinson, A. Lewis, G. H. Lewis, A. M. Leyko, M. Leyton, B. Li, B. Li, H. Li, H. L. Li, S. Li, X. Li, Z. Liang, H. Liao, B. Liberti, P. Lichard, K. Lie, J. Liebal, W. Liebig, C. Limbach, A. Limosani, M. Limper, S. C. Lin, F. Linde, J. T. Linnemann, E. Lipeles, A. Lipniacka, M. Lisovyi, T. M. Liss, D. Lissauer, A. Lister, A. M. Litke, D. Liu, J. B. Liu, L. Liu, M. Liu, Y. Liu, M. Livan, S. S. A. Livermore, A. Lleres, J. Llorente Merino, S. L. Lloyd, F. Lo Sterzo, E. Lobodzinska, P. Loch, W. S. Lockman, T. Loddenkoetter, F. K. Loebinger, A. E. Loevschall-Jensen, A. Loginov, C. W. Loh, T. Lohse, K. Lohwasser, M. Lokajicek, V. P. Lombardo, R. E. Long, L. Lopes, D. Lopez Mateos, J. Lorenz, N. Lorenzo Martinez, M. Losada, P. Loscutoff, M. J. Losty, X. Lou, A. Lounis, K. F. Loureiro, J. Love, P. A. Love, A. J. Lowe, F. Lu, H. J. Lubatti, C. Luci, A. Lucotte, D. Ludwig, I. Ludwig, J. Ludwig, F. Luehring, W. Lukas, L. Luminari, E. Lund, B. Lundberg, J. Lundberg, O. Lundberg, B. Lund-Jensen, J. Lundquist, M. Lungwitz, D. Lynn, R. Lysak, E. Lytken, H. Ma, L. L. Ma, G. Maccarrone, A. Macchiolo, B. Maček, J. Machado Miguens, D. Macina, R. Mackeprang, R. Madar, R. J. Madaras, H. J. Maddocks, W. F. Mader, A. Madsen, M. Maeno, T. Maeno, L. Magnoni, E. Magradze, K. Mahboubi, J. Mahlstedt, S. Mahmoud, G. Mahout, C. Maiani, C. Maidantchik, A. Maio, S. Majewski, Y. Makida, N. Makovec, P. Mal, B. Malaescu, Pa. Malecki, P. Malecki, V. P. Maleev, F. Malek, U. Mallik, D. Malon, C. Malone, S. Maltezos, V. Malyshev, S. Malyukov, J. Mamuzic, A. Manabe, L. Mandelli, I. Mandić, R. Mandrysch, J. Maneira, A. Manfredini, L. Manhaes de Andrade Filho, J. A. Manjarres Ramos, A. Mann, P. M. Manning, A. Manousakis-Katsikakis, B. Mansoulie, R. Mantifel, A. Mapelli, L. Mapelli, L. March, J. F. Marchand, F. Marchese, G. Marchiori, M. Marcisovsky, C. P. Marino, F. Marroquim, Z. Marshall, L. F. Marti, S. Marti-Garcia, B. Martin, B. Martin, J. P. Martin, T. A. Martin, V. J. Martin, B. Martin dit Latour, H. Martinez, M. Martinez, V. Martinez Outschoorn, S. Martin-Haugh, A. C. Martyniuk, M. Marx, F. Marzano, A. Marzin, L. Masetti, T. Mashimo, R. Mashinistov, J. Masik, A. L. Maslennikov, I. Massa, N. Massol, P. Mastrandrea, A. Mastroberardino, T. Masubuchi, H. Matsunaga, T. Matsushita, P. Mättig, S. Mättig, C. Mattravers, J. Maurer, S. J. Maxfield, D. A. Maximov, R. Mazini, M. Mazur, L. Mazzaferro, M. Mazzanti, J. Mc Donald, S. P. Mc Kee, A. McCarn, R. L. McCarthy, T. G. McCarthy, N. A. McCubbin, K. W. McFarlane, J. A. Mcfayden, G. Mchedlidze, T. Mclaughlan, S. J. McMahon, R. A. McPherson, A. Meade, J. Mechnich, M. Mechtel, M. Medinnis, S. Meehan, R. Meera-Lebbai, T. Meguro, S. Mehlhase, A. Mehta, K. Meier, C. Meineck, B. Meirose, C. Melachrinos, B. R. Mellado Garcia, F. Meloni, L. Mendoza Navas, Z. Meng, A. Mengarelli, S. Menke, E. Meoni, K. M. Mercurio, N. Meric, P. Mermod, L. Merola, C. Meroni, F. S. Merritt, H. Merritt, A. Messina, J. Metcalfe, A. S. Mete, C. Meyer, C. Meyer, J-P. Meyer, J. Meyer, J. Meyer, S. Michal, L. Micu, R. P. Middleton, S. Migas, L. Mijović, G. Mikenberg, M. Mikestikova, M. Mikuž, D. W. Miller, R. J. Miller, W. J. Mills, C. Mills, A. Milov, D. A. Milstead, D. Milstein, A. A. Minaenko, M. Miñano Moya, I. A. Minashvili, A. I. Mincer, B. Mindur, M. Mineev, Y. Ming, L. M. Mir, G. Mirabelli, J. Mitrevski, V. A. Mitsou, S. Mitsui, P. S. Miyagawa, J. U. Mjörnmark, T. Moa, V. Moeller, S. Mohapatra, W. Mohr, R. Moles-Valls, A. Molfetas, K. Mönig, C. Monini, J. Monk, E. Monnier, J. Montejo Berlingen, F. Monticelli, S. Monzani, R. W. Moore, C. Mora Herrera, A. Moraes, N. Morange, J. Morel, D. Moreno, M. Moreno Llácer, P. Morettini, M. Morgenstern, M. Morii, A. K. Morley, G. Mornacchi, J. D. Morris, L. Morvaj, N. Möser, H. G. Moser, M. Mosidze, J. Moss, R. Mount, E. Mountricha, S. V. Mouraviev, E. J. W. Moyse, F. Mueller, J. Mueller, K. Mueller, T. Mueller, D. Muenstermann, T. A. Müller, Y. Munwes, W. J. Murray, I. Mussche, E. Musto, A. G. Myagkov, M. Myska, O. Nackenhorst, J. Nadal, K. Nagai, R. Nagai, Y. Nagai, K. Nagano, A. Nagarkar, Y. Nagasaka, M. Nagel, A. M. Nairz, Y. Nakahama, K. Nakamura, T. Nakamura, I. Nakano, H. Namasivayam, G. Nanava, A. Napier, R. Narayan, M. Nash, T. Nattermann, T. Naumann, G. Navarro, H. A. Neal, P. Yu. Nechaeva, T. J. Neep, A. Negri, G. Negri, M. Negrini, S. Nektarijevic, A. Nelson, T. K. Nelson, S. Nemecek, P. Nemethy, A. A. Nepomuceno, M. Nessi, M. S. Neubauer, M. Neumann, A. Neusiedl, R. M. Neves, P. Nevski, F. M. Newcomer, P. R. Newman, D. H. Nguyen, V. Nguyen Thi Hong, R. B. Nickerson, R. Nicolaidou, B. Nicquevert, F. Niedercorn, J. Nielsen, N. Nikiforou, A. Nikiforov, V. Nikolaenko, I. Nikolic-Audit, K. Nikolics, K. Nikolopoulos, H. Nilsen, P. Nilsson, Y. Ninomiya, A. Nisati, R. Nisius, T. Nobe, L. Nodulman, M. Nomachi, I. Nomidis, S. Norberg, M. Nordberg, J. Novakova, M. Nozaki, L. Nozka, A.-E. Nuncio-Quiroz, G. Nunes Hanninger, T. Nunnemann, E. Nurse, B. J. O’Brien, D. C. O’Neil, V. O’Shea, L. B. Oakes, F. G. Oakham, H. Oberlack, J. Ocariz, A. Ochi, M. I. Ochoa, S. Oda, S. Odaka, J. Odier, H. Ogren, A. Oh, S. H. Oh, C. C. Ohm, T. Ohshima, W. Okamura, H. Okawa, Y. Okumura, T. Okuyama, A. Olariu, A. G. Olchevski, S. A. Olivares Pino, M. Oliveira, D. Oliveira Damazio, E. Oliver Garcia, D. Olivito, A. Olszewski, J. Olszowska, A. Onofre, P. U. E. Onyisi, C. J. Oram, M. J. Oreglia, Y. Oren, D. Orestano, N. Orlando, C. Oropeza Barrera, R. S. Orr, B. Osculati, R. Ospanov, C. Osuna, G. Otero y Garzon, J. P. Ottersbach, M. Ouchrif, E. A. Ouellette, F. Ould-Saada, A. Ouraou, Q. Ouyang, A. Ovcharova, M. Owen, S. Owen, V. E. Ozcan, N. Ozturk, A. Pacheco Pages, C. Padilla Aranda, S. Pagan Griso, E. Paganis, C. Pahl, F. Paige, P. Pais, K. Pajchel, G. Palacino, C. P. Paleari, S. Palestini, D. Pallin, A. Palma, J. D. Palmer, Y. B. Pan, E. Panagiotopoulou, J. G. Panduro Vazquez, P. Pani, N. Panikashvili, S. Panitkin, D. Pantea, A. Papadelis, Th. D. Papadopoulou, A. Paramonov, D. Paredes Hernandez, W. Park, M. A. Parker, F. Parodi, J. A. Parsons, U. Parzefall, S. Pashapour, E. Pasqualucci, S. Passaggio, A. Passeri, F. Pastore, Fr. Pastore, G. Pásztor, S. Pataraia, N. D. Patel, J. R. Pater, S. Patricelli, T. Pauly, J. Pearce, M. Pedersen, S. Pedraza Lopez, M. I. Pedraza Morales, S. V. Peleganchuk, D. Pelikan, H. Peng, B. Penning, A. Penson, J. Penwell, T. Perez Cavalcanti, E. Perez Codina, M. T. Pérez García-Estañ, V. Perez Reale, L. Perini, H. Pernegger, R. Perrino, P. Perrodo, V. D. Peshekhonov, K. Peters, R. F. Y. Peters, B. A. Petersen, J. Petersen, T. C. Petersen, E. Petit, A. Petridis, C. Petridou, E. Petrolo, F. Petrucci, D. Petschull, M. Petteni, R. Pezoa, A. Phan, P. W. Phillips, G. Piacquadio, E. Pianori, A. Picazio, E. Piccaro, M. Piccinini, S. M. Piec, R. Piegaia, D. T. Pignotti, J. E. Pilcher, A. D. Pilkington, J. Pina, M. Pinamonti, A. Pinder, J. L. Pinfold, A. Pingel, B. Pinto, C. Pizio, M.-A. Pleier, V. Pleskot, E. Plotnikova, P. Plucinski, A. Poblaguev, S. Poddar, F. Podlyski, R. Poettgen, L. Poggioli, D. Pohl, M. Pohl, G. Polesello, A. Policicchio, R. Polifka, A. Polini, J. Poll, V. Polychronakos, D. Pomeroy, K. Pommès, L. Pontecorvo, B. G. Pope, G. A. Popeneciu, D. S. Popovic, A. Poppleton, X. Portell Bueso, G. E. Pospelov, S. Pospisil, I. N. Potrap, C. J. Potter, C. T. Potter, G. Poulard, J. Poveda, V. Pozdnyakov, R. Prabhu, P. Pralavorio, A. Pranko, S. Prasad, R. Pravahan, S. Prell, K. Pretzl, D. Price, J. Price, L. E. Price, D. Prieur, M. Primavera, M. Proissl, K. Prokofiev, F. Prokoshin, E. Protopapadaki, S. Protopopescu, J. Proudfoot, X. Prudent, M. Przybycien, H. Przysiezniak, S. Psoroulas, E. Ptacek, E. Pueschel, D. Puldon, M. Purohit, P. Puzo, Y. Pylypchenko, J. Qian, A. Quadt, D. R. Quarrie, W. B. Quayle, D. Quilty, M. Raas, V. Radeka, V. Radescu, P. Radloff, F. Ragusa, G. Rahal, A. M. Rahimi, S. Rajagopalan, M. Rammensee, M. Rammes, A. S. Randle-Conde, K. Randrianarivony, C. Rangel-Smith, K. Rao, F. Rauscher, T. C. Rave, T. Ravenscroft, M. Raymond, A. L. Read, D. M. Rebuzzi, A. Redelbach, G. Redlinger, R. Reece, K. Reeves, A. Reinsch, I. Reisinger, M. Relich, C. Rembser, Z. L. Ren, A. Renaud, M. Rescigno, S. Resconi, B. Resende, P. Reznicek, R. Rezvani, R. Richter, E. Richter-Was, M. Ridel, P. Rieck, M. Rijssenbeek, A. Rimoldi, L. Rinaldi, R. R. Rios, E. Ritsch, I. Riu, G. Rivoltella, F. Rizatdinova, E. Rizvi, S. H. Robertson, A. Robichaud-Veronneau, D. Robinson, J. E. M. Robinson, A. Robson, J. G. Rocha de Lima, C. Roda, D. Roda Dos Santos, A. Roe, S. Roe, O. Røhne, S. Rolli, A. Romaniouk, M. Romano, G. Romeo, E. Romero Adam, N. Rompotis, L. Roos, E. Ros, S. Rosati, K. Rosbach, A. Rose, M. Rose, G. A. Rosenbaum, P. L. Rosendahl, O. Rosenthal, L. Rosselet, V. Rossetti, E. Rossi, L. P. Rossi, M. Rotaru, I. Roth, J. Rothberg, D. Rousseau, C. R. Royon, A. Rozanov, Y. Rozen, X. Ruan, F. Rubbo, I. Rubinskiy, N. Ruckstuhl, V. I. Rud, C. Rudolph, M. S. Rudolph, F. Rühr, A. Ruiz-Martinez, L. Rumyantsev, Z. Rurikova, N. A. Rusakovich, A. Ruschke, J. P. Rutherfoord, N. Ruthmann, P. Ruzicka, Y. F. Ryabov, M. Rybar, G. Rybkin, N. C. Ryder, A. F. Saavedra, I. Sadeh, H. F-W. Sadrozinski, R. Sadykov, F. Safai Tehrani, H. Sakamoto, G. Salamanna, A. Salamon, M. Saleem, D. Salek, D. Salihagic, A. Salnikov, J. Salt, B. M. Salvachua Ferrando, D. Salvatore, F. Salvatore, A. Salvucci, A. Salzburger, D. Sampsonidis, A. Sanchez, J. Sánchez, V. Sanchez Martinez, H. Sandaker, H. G. Sander, M. P. Sanders, M. Sandhoff, T. Sandoval, C. Sandoval, R. Sandstroem, D. P. C. Sankey, A. Sansoni, C. Santamarina Rios, C. Santoni, R. Santonico, H. Santos, I. Santoyo Castillo, K. Sapp, J. G. Saraiva, T. Sarangi, E. Sarkisyan-Grinbaum, B. Sarrazin, F. Sarri, G. Sartisohn, O. Sasaki, Y. Sasaki, N. Sasao, I. Satsounkevitch, G. Sauvage, E. Sauvan, J. B. Sauvan, P. Savard, V. Savinov, D. O. Savu, L. Sawyer, D. H. Saxon, J. Saxon, C. Sbarra, A. Sbrizzi, D. A. Scannicchio, M. Scarcella, J. Schaarschmidt, P. Schacht, D. Schaefer, A. Schaelicke, S. Schaepe, S. Schaetzel, U. Schäfer, A. C. Schaffer, D. Schaile, R. D. Schamberger, V. Scharf, V. A. Schegelsky, D. Scheirich, M. Schernau, M. I. Scherzer, C. Schiavi, J. Schieck, C. Schillo, M. Schioppa, S. Schlenker, E. Schmidt, K. Schmieden, C. Schmitt, C. Schmitt, S. Schmitt, B. Schneider, Y. J. Schnellbach, U. Schnoor, L. Schoeffel, A. Schoening, A. L. S. Schorlemmer, M. Schott, D. Schouten, J. Schovancova, M. Schram, C. Schroeder, N. Schroer, M. J. Schultens, J. Schultes, H.-C. Schultz-Coulon, H. Schulz, M. Schumacher, B. A. Schumm, Ph. Schune, A. Schwartzman, Ph. Schwegler, Ph. Schwemling, R. Schwienhorst, J. Schwindling, T. Schwindt, M. Schwoerer, F. G. Sciacca, E. Scifo, G. Sciolla, W. G. Scott, J. Searcy, G. Sedov, E. Sedykh, S. C. Seidel, A. Seiden, F. Seifert, J. M. Seixas, G. Sekhniaidze, S. J. Sekula, K. E. Selbach, D. M. Seliverstov, B. Sellden, G. Sellers, M. Seman, N. Semprini-Cesari, C. Serfon, L. Serin, L. Serkin, T. Serre, R. Seuster, H. Severini, A. Sfyrla, E. Shabalina, M. Shamim, L. Y. Shan, J. T. Shank, Q. T. Shao, M. Shapiro, P. B. Shatalov, K. Shaw, P. Sherwood, S. Shimizu, M. Shimojima, T. Shin, M. Shiyakova, A. Shmeleva, M. J. Shochet, D. Short, S. Shrestha, E. Shulga, M. A. Shupe, P. Sicho, A. Sidoti, F. Siegert, Dj. Sijacki, O. Silbert, J. Silva, Y. Silver, D. Silverstein, S. B. Silverstein, V. Simak, O. Simard, Lj. Simic, S. Simion, E. Simioni, B. Simmons, R. Simoniello, M. Simonyan, P. Sinervo, N. B. Sinev, V. Sipica, G. Siragusa, A. Sircar, A. N. Sisakyan, S. Yu. Sivoklokov, J. Sjölin, T. B. Sjursen, L. A. Skinnari, H. P. Skottowe, K. Skovpen, P. Skubic, M. Slater, T. Slavicek, K. Sliwa, V. Smakhtin, B. H. Smart, L. Smestad, S. Yu. Smirnov, Y. Smirnov, L. N. Smirnova, O. Smirnova, B. C. Smith, K. M. Smith, M. Smizanska, K. Smolek, A. A. Snesarev, G. Snidero, S. W. Snow, J. Snow, S. Snyder, R. Sobie, J. Sodomka, A. Soffer, D. A. Soh, C. A. Solans, M. Solar, J. Solc, E. Yu. Soldatov, U. Soldevila, E. Solfaroli Camillocci, A. A. Solodkov, O. V. Solovyanov, V. Solovyev, N. Soni, A. Sood, V. Sopko, B. Sopko, M. Sosebee, R. Soualah, P. Soueid, A. Soukharev, D. South, S. Spagnolo, F. Spanò, R. Spighi, G. Spigo, R. Spiwoks, M. Spousta, T. Spreitzer, B. Spurlock, R. D. St. Denis, J. Stahlman, R. Stamen, E. Stanecka, R. W. Stanek, C. Stanescu, M. Stanescu-Bellu, M. M. Stanitzki, S. Stapnes, E. A. Starchenko, J. Stark, P. Staroba, P. Starovoitov, R. Staszewski, A. Staude, P. Stavina, G. Steele, P. Steinbach, P. Steinberg, I. Stekl, B. Stelzer, H. J. Stelzer, O. Stelzer-Chilton, H. Stenzel, S. Stern, G. A. Stewart, J. A. Stillings, M. C. Stockton, M. Stoebe, K. Stoerig, G. Stoicea, S. Stonjek, P. Strachota, A. R. Stradling, A. Straessner, J. Strandberg, S. Strandberg, A. Strandlie, M. Strang, E. Strauss, M. Strauss, P. Strizenec, R. Ströhmer, D. M. Strom, J. A. Strong, R. Stroynowski, B. Stugu, I. Stumer, J. Stupak, P. Sturm, N. A. Styles, D. Su, HS. Subramania, R. Subramaniam, A. Succurro, Y. Sugaya, C. Suhr, M. Suk, V. V. Sulin, S. Sultansoy, T. Sumida, X. Sun, J. E. Sundermann, K. Suruliz, G. Susinno, M. R. Sutton, Y. Suzuki, Y. Suzuki, M. Svatos, S. Swedish, M. Swiatlowski, I. Sykora, T. Sykora, D. Ta, K. Tackmann, A. Taffard, R. Tafirout, N. Taiblum, Y. Takahashi, H. Takai, R. Takashima, H. Takeda, T. Takeshita, Y. Takubo, M. Talby, A. Talyshev, J. Y. C. Tam, M. C. Tamsett, K. G. Tan, J. Tanaka, R. Tanaka, S. Tanaka, S. Tanaka, A. J. Tanasijczuk, K. Tani, N. Tannoury, S. Tapprogge, D. Tardif, S. Tarem, F. Tarrade, G. F. Tartarelli, P. Tas, M. Tasevsky, E. Tassi, Y. Tayalati, C. Taylor, F. E. Taylor, G. N. Taylor, W. Taylor, M. Teinturier, F. A. Teischinger, M. Teixeira Dias Castanheira, P. Teixeira-Dias, K. K. Temming, H. Ten Kate, P. K. Teng, S. Terada, K. Terashi, J. Terron, M. Testa, R. J. Teuscher, J. Therhaag, T. Theveneaux-Pelzer, S. Thoma, J. P. Thomas, E. N. Thompson, P. D. Thompson, P. D. Thompson, A. S. Thompson, L. A. Thomsen, E. Thomson, M. Thomson, W. M. Thong, R. P. Thun, F. Tian, M. J. Tibbetts, T. Tic, V. O. Tikhomirov, Y. A. Tikhonov, S. Timoshenko, E. Tiouchichine, P. Tipton, S. Tisserant, T. Todorov, S. Todorova-Nova, B. Toggerson, J. Tojo, S. Tokár, K. Tokushuku, K. Tollefson, L. Tomlinson, M. Tomoto, L. Tompkins, K. Toms, A. Tonoyan, C. Topfel, N. D. Topilin, E. Torrence, H. Torres, E. Torró Pastor, J. Toth, F. Touchard, D. R. Tovey, H. L. Tran, T. Trefzger, L. Tremblet, A. Tricoli, I. M. Trigger, S. Trincaz-Duvoid, M. F. Tripiana, N. Triplett, W. Trischuk, B. Trocmé, C. Troncon, M. Trottier-McDonald, M. Trovatelli, P. True, M. Trzebinski, A. Trzupek, C. Tsarouchas, J. C-L. Tseng, M. Tsiakiris, P. V. Tsiareshka, D. Tsionou, G. Tsipolitis, S. Tsiskaridze, V. Tsiskaridze, E. G. Tskhadadze, I. I. Tsukerman, V. Tsulaia, J.-W. Tsung, S. Tsuno, D. Tsybychev, A. Tua, A. Tudorache, V. Tudorache, J. M. Tuggle, M. Turala, D. Turecek, I. Turk Cakir, R. Turra, P. M. Tuts, A. Tykhonov, M. Tylmad, M. Tyndel, G. Tzanakos, K. Uchida, I. Ueda, R. Ueno, M. Ughetto, M. Ugland, M. Uhlenbrock, F. Ukegawa, G. Unal, A. Undrus, G. Unel, F. C. Ungaro, Y. Unno, D. Urbaniec, P. Urquijo, G. Usai, L. Vacavant, V. Vacek, B. Vachon, S. Vahsen, N. Valencic, S. Valentinetti, A. Valero, L. Valery, S. Valkar, E. Valladolid Gallego, S. Vallecorsa, J. A. Valls Ferrer, R. Van Berg, P. C. Van Der Deijl, R. van der Geer, H. van der Graaf, R. Van Der Leeuw, E. van der Poel, D. van der Ster, N. van Eldik, P. van Gemmeren, J. Van Nieuwkoop, I. van Vulpen, M. Vanadia, W. Vandelli, A. Vaniachine, P. Vankov, F. Vannucci, R. Vari, E. W. Varnes, T. Varol, D. Varouchas, A. Vartapetian, K. E. Varvell, V. I. Vassilakopoulos, F. Vazeille, T. Vazquez Schroeder, F. Veloso, S. Veneziano, A. Ventura, D. Ventura, M. Venturi, N. Venturi, V. Vercesi, M. Verducci, W. Verkerke, J. C. Vermeulen, A. Vest, M. C. Vetterli, I. Vichou, T. Vickey, O. E. Vickey Boeriu, G. H. A. Viehhauser, S. Viel, M. Villa, M. Villaplana Perez, E. Vilucchi, M. G. Vincter, E. Vinek, V. B. Vinogradov, J. Virzi, O. Vitells, M. Viti, I. Vivarelli, F. Vives Vaque, S. Vlachos, D. Vladoiu, M. Vlasak, A. Vogel, P. Vokac, G. Volpi, M. Volpi, G. Volpini, H. von der Schmitt, H. von Radziewski, E. von Toerne, V. Vorobel, V. Vorwerk, M. Vos, R. Voss, J. H. Vossebeld, N. Vranjes, M. Vranjes Milosavljevic, V. Vrba, M. Vreeswijk, T. Vu Anh, R. Vuillermet, I. Vukotic, Z. Vykydal, W. Wagner, P. Wagner, H. Wahlen, S. Wahrmund, J. Wakabayashi, S. Walch, J. Walder, R. Walker, W. Walkowiak, R. Wall, P. Waller, B. Walsh, C. Wang, H. Wang, H. Wang, J. Wang, J. Wang, K. Wang, R. Wang, S. M. Wang, T. Wang, X. Wang, A. Warburton, C. P. Ward, D. R. Wardrope, M. Warsinsky, A. Washbrook, C. Wasicki, I. Watanabe, P. M. Watkins, A. T. Watson, I. J. Watson, M. F. Watson, G. Watts, S. Watts, A. T. Waugh, B. M. Waugh, M. S. Weber, J. S. Webster, A. R. Weidberg, P. Weigell, J. Weingarten, C. Weiser, P. S. Wells, T. Wenaus, D. Wendland, Z. Weng, T. Wengler, S. Wenig, N. Wermes, M. Werner, P. Werner, M. Werth, M. Wessels, J. Wetter, C. Weydert, K. Whalen, A. White, M. J. White, S. White, S. R. Whitehead, D. Whiteson, D. Whittington, D. Wicke, F. J. Wickens, W. Wiedenmann, M. Wielers, P. Wienemann, C. Wiglesworth, L. A. M. Wiik-Fuchs, P. A. Wijeratne, A. Wildauer, M. A. Wildt, I. Wilhelm, H. G. Wilkens, J. Z. Will, E. Williams, H. H. Williams, S. Williams, W. Willis, S. Willocq, J. A. Wilson, M. G. Wilson, A. Wilson, I. Wingerter-Seez, S. Winkelmann, F. Winklmeier, M. Wittgen, T. Wittig, J. Wittkowski, S. J. Wollstadt, M. W. Wolter, H. Wolters, W. C. Wong, G. Wooden, B. K. Wosiek, J. Wotschack, M. J. Woudstra, K. W. Wozniak, K. Wraight, M. Wright, B. Wrona, S. L. Wu, X. Wu, Y. Wu, E. Wulf, B. M. Wynne, S. Xella, M. Xiao, S. Xie, C. Xu, D. Xu, L. Xu, B. Yabsley, S. Yacoob, M. Yamada, H. Yamaguchi, Y. Yamaguchi, A. Yamamoto, K. Yamamoto, S. Yamamoto, T. Yamamura, T. Yamanaka, K. Yamauchi, T. Yamazaki, Y. Yamazaki, Z. Yan, H. Yang, H. Yang, U. K. Yang, Y. Yang, Z. Yang, S. Yanush, L. Yao, Y. Yasu, E. Yatsenko, J. Ye, S. Ye, A. L. Yen, M. Yilmaz, R. Yoosoofmiya, K. Yorita, R. Yoshida, K. Yoshihara, C. Young, C. J. S. Young, S. Youssef, D. Yu, D. R. Yu, J. Yu, J. Yu, L. Yuan, A. Yurkewicz, B. Zabinski, R. Zaidan, A. M. Zaitsev, S. Zambito, L. Zanello, D. Zanzi, A. Zaytsev, C. Zeitnitz, M. Zeman, A. Zemla, O. Zenin, T. Ženiš, D. Zerwas, G. Zevi della Porta, D. Zhang, H. Zhang, J. Zhang, L. Zhang, X. Zhang, Z. Zhang, L. Zhao, Z. Zhao, A. Zhemchugov, J. Zhong, B. Zhou, N. Zhou, Y. Zhou, C. G. Zhu, H. Zhu, J. Zhu, Y. Zhu, X. Zhuang, V. Zhuravlov, A. Zibell, D. Zieminska, N. I. Zimin, R. Zimmermann, S. Zimmermann, S. Zimmermann, Z. Zinonos, M. Ziolkowski, R. Zitoun, L. Živković, V. V. Zmouchko, G. Zobernig, A. Zoccoli, M. zur Nedden, V. Zutshi, L. Zwalinski

**Affiliations:** 1 CERN, 1211 Geneva 23, Switzerland; 2 School of Chemistry and Physics, University of Adelaide, Adelaide, Australia; 3 Physics Department, SUNY Albany, Albany, NY United States of America; 4 Department of Physics, University of Alberta, Edmonton, AB Canada; 5 Department of Physics, Ankara University, Ankara, Turkey; 6 Department of Physics, Dumlupinar University, Kutahya, Turkey; 7 Department of Physics, Gazi University, Ankara, Turkey; 8 Division of Physics, TOBB University of Economics and Technology, Ankara, Turkey; 9 Turkish Atomic Energy Authority, Ankara, Turkey; 10 LAPP, CNRS/IN2P3 and Université de Savoie, Annecy-le-Vieux, France; 11 High Energy Physics Division, Argonne National Laboratory, Argonne, IL United States of America; 12 Department of Physics, University of Arizona, Tucson, AZ United States of America; 13 Department of Physics, The University of Texas at Arlington, Arlington, TX United States of America; 14 Physics Department, University of Athens, Athens, Greece; 15 Physics Department, National Technical University of Athens, Zografou, Greece; 16 Institute of Physics, Azerbaijan Academy of Sciences, Baku, Azerbaijan; 17 Institut de Física d’Altes Energies and Departament de Física de la Universitat Autònoma de Barcelona, and ICREA, Barcelona, Spain; 18 Institute of Physics, University of Belgrade, Belgrade, Serbia; 19 Vinca Institute of Nuclear Sciences, University of Belgrade, Belgrade, Serbia; 20 Department for Physics and Technology, University of Bergen, Bergen, Norway; 21 Physics Division, Lawrence Berkeley National Laboratory and University of California, Berkeley, CA United States of America; 22 Department of Physics, Humboldt University, Berlin, Germany; 23 Albert Einstein Center for Fundamental Physics and Laboratory for High Energy Physics, University of Bern, Bern, Switzerland; 24 School of Physics and Astronomy, University of Birmingham, Birmingham, United Kingdom; 25 Department of Physics, Bogazici University, Istanbul, Turkey; 26 Division of Physics, Dogus University, Istanbul, Turkey; 27 Department of Physics Engineering, Gaziantep University, Gaziantep, Turkey; 28 Department of Physics, Istanbul Technical University, Istanbul, Turkey; 29 INFN Sezione di Bologna, Bologna, Italy; 30 Dipartimento di Fisica, Università di Bologna, Bologna, Italy; 31 Physikalisches Institut, University of Bonn, Bonn, Germany; 32 Department of Physics, Boston University, Boston, MA United States of America; 33 Department of Physics, Brandeis University, Waltham, MA United States of America; 34 Universidade Federal do Rio De Janeiro COPPE/EE/IF, Rio de Janeiro, Brazil; 35 Federal University of Juiz de Fora (UFJF), Juiz de Fora, Brazil; 36 Federal University of Sao Joao del Rei (UFSJ), Sao Joao del Rei, Brazil; 37 Instituto de Fisica, Universidade de Sao Paulo, Sao Paulo, Brazil; 38 Physics Department, Brookhaven National Laboratory, Upton, NY United States of America; 39 National Institute of Physics and Nuclear Engineering, Bucharest, Romania; 40 University Politehnica Bucharest, Bucharest, Romania; 41 West University in Timisoara, Timisoara, Romania; 42 Departamento de Física, Universidad de Buenos Aires, Buenos Aires, Argentina; 43 Cavendish Laboratory, University of Cambridge, Cambridge, United Kingdom; 44 Department of Physics, Carleton University, Ottawa, ON Canada; 45 CERN, Geneva, Switzerland; 46 Enrico Fermi Institute, University of Chicago, Chicago, IL United States of America; 47 Departamento de Física, Pontificia Universidad Católica de Chile, Santiago, Chile; 48 Departamento de Física, Universidad Técnica Federico Santa María, Valparaíso, Chile; 49 Institute of High Energy Physics, Chinese Academy of Sciences, Beijing, China; 50 Department of Modern Physics, University of Science and Technology of China, Anhui, China; 51 Department of Physics, Nanjing University, Jiangsu, China; 52 School of Physics, Shandong University, Shandong, China; 53 Physics Department, Shanghai Jiao Tong University, Shanghai, China; 54 Laboratoire de Physique Corpusculaire, Clermont Université and Université Blaise Pascal and CNRS/IN2P3, Clermont-Ferrand, France; 55 Nevis Laboratory, Columbia University, Irvington, NY United States of America; 56 Niels Bohr Institute, University of Copenhagen, Kobenhavn, Denmark; 57 INFN Gruppo Collegato di Cosenza, Rende, Italy; 58 Dipartimento di Fisica, Università della Calabria, Rende, Italy; 59 AGH University of Science and Technology, Faculty of Physics and Applied Computer Science, Krakow, Poland; 60 The Henryk Niewodniczanski Institute of Nuclear Physics, Polish Academy of Sciences, Krakow, Poland; 61 Physics Department, Southern Methodist University, Dallas, TX United States of America; 62 Physics Department, University of Texas at Dallas, Richardson, TX United States of America; 63 DESY, Hamburg and Zeuthen, Germany; 64 Institut für Experimentelle Physik IV, Technische Universität Dortmund, Dortmund, Germany; 65 Institut für Kern- und Teilchenphysik, Technical University Dresden, Dresden, Germany; 66 Department of Physics, Duke University, Durham, NC United States of America; 67 SUPA - School of Physics and Astronomy, University of Edinburgh, Edinburgh, United Kingdom; 68 INFN Laboratori Nazionali di Frascati, Frascati, Italy; 69 Fakultät für Mathematik und Physik, Albert-Ludwigs-Universität, Freiburg, Germany; 70 Section de Physique, Université de Genève, Geneva, Switzerland; 71 INFN Sezione di Genova, Genova, Italy; 72 Dipartimento di Fisica, Università di Genova, Genova, Italy; 73 E. Andronikashvili Institute of Physics, Iv. Javakhishvili Tbilisi State University, Tbilisi, Georgia; 74 High Energy Physics Institute, Tbilisi State University, Tbilisi, Georgia; 75 II Physikalisches Institut, Justus-Liebig-Universität Giessen, Giessen, Germany; 76 SUPA - School of Physics and Astronomy, University of Glasgow, Glasgow, United Kingdom; 77 II Physikalisches Institut, Georg-August-Universität, Göttingen, Germany; 78 Laboratoire de Physique Subatomique et de Cosmologie, Université Joseph Fourier and CNRS/IN2P3 and Institut National Polytechnique de Grenoble, Grenoble, France; 79 Department of Physics, Hampton University, Hampton, VA United States of America; 80 Laboratory for Particle Physics and Cosmology, Harvard University, Cambridge, MA United States of America; 81 Kirchhoff-Institut für Physik, Ruprecht-Karls-Universität Heidelberg, Heidelberg, Germany; 82 Physikalisches Institut, Ruprecht-Karls-Universität Heidelberg, Heidelberg, Germany; 83 ZITI Institut für technische Informatik, Ruprecht-Karls-Universität Heidelberg, Mannheim, Germany; 84 Faculty of Applied Information Science, Hiroshima Institute of Technology, Hiroshima, Japan; 85 Department of Physics, Indiana University, Bloomington, IN United States of America; 86 Institut für Astro- und Teilchenphysik, Leopold-Franzens-Universität, Innsbruck, Austria; 87 University of Iowa, Iowa City, IA United States of America; 88 Department of Physics and Astronomy, Iowa State University, Ames, IA United States of America; 89 Joint Institute for Nuclear Research, JINR Dubna, Dubna, Russia; 90 KEK, High Energy Accelerator Research Organization, Tsukuba, Japan; 91 Graduate School of Science, Kobe University, Kobe, Japan; 92 Faculty of Science, Kyoto University, Kyoto, Japan; 93 Kyoto University of Education, Kyoto, Japan; 94 Department of Physics, Kyushu University, Fukuoka, Japan; 95 Instituto de Física La Plata, Universidad Nacional de La Plata and CONICET, La Plata, Argentina; 96 Physics Department, Lancaster University, Lancaster, United Kingdom; 97 INFN Sezione di Lecce, Lecce, Italy; 98 Dipartimento di Matematica e Fisica, Università del Salento, Lecce, Italy; 99 Oliver Lodge Laboratory, University of Liverpool, Liverpool, United Kingdom; 100 Department of Physics, Jožef Stefan Institute and University of Ljubljana, Ljubljana, Slovenia; 101 School of Physics and Astronomy, Queen Mary University of London, London, United Kingdom; 102 Department of Physics, Royal Holloway University of London, Surrey, United Kingdom; 103 Department of Physics and Astronomy, University College London, London, United Kingdom; 104 Laboratoire de Physique Nucléaire et de Hautes Energies, UPMC and Université Paris-Diderot and CNRS/IN2P3, Paris, France; 105 Fysiska institutionen, Lunds universitet, Lund, Sweden; 106 Departamento de Fisica Teorica C-15, Universidad Autonoma de Madrid, Madrid, Spain; 107 Institut für Physik, Universität Mainz, Mainz, Germany; 108 School of Physics and Astronomy, University of Manchester, Manchester, United Kingdom; 109 CPPM, Aix-Marseille Université and CNRS/IN2P3, Marseille, France; 110 Department of Physics, University of Massachusetts, Amherst, MA United States of America; 111 Department of Physics, McGill University, Montreal, QC Canada; 112 School of Physics, University of Melbourne, Victoria, Australia; 113 Department of Physics, The University of Michigan, Ann Arbor, MI United States of America; 114 Department of Physics and Astronomy, Michigan State University, East Lansing, MI United States of America; 115 INFN Sezione di Milano, Milano, Italy; 116 Dipartimento di Fisica, Università di Milano, Milano, Italy; 117 B.I. Stepanov Institute of Physics, National Academy of Sciences of Belarus, Minsk, Republic of Belarus; 118 National Scientific and Educational Centre for Particle and High Energy Physics, Minsk, Republic of Belarus; 119 Department of Physics, Massachusetts Institute of Technology, Cambridge, MA United States of America; 120 Group of Particle Physics, University of Montreal, Montreal, QC Canada; 121 P.N. Lebedev Institute of Physics, Academy of Sciences, Moscow, Russia; 122 Institute for Theoretical and Experimental Physics (ITEP), Moscow, Russia; 123 Moscow Engineering and Physics Institute (MEPhI), Moscow, Russia; 124 D.V.Skobeltsyn Institute of Nuclear Physics, M.V.Lomonosov Moscow State University, Moscow, Russia; 125 Fakultät für Physik, Ludwig-Maximilians-Universität München, München, Germany; 126 Max-Planck-Institut für Physik (Werner-Heisenberg-Institut), München, Germany; 127 Nagasaki Institute of Applied Science, Nagasaki, Japan; 128 Graduate School of Science and Kobayashi-Maskawa Institute, Nagoya University, Nagoya, Japan; 129 INFN Sezione di Napoli, Napoli, Italy; 130 Dipartimento di Scienze Fisiche, Università di Napoli, Napoli, Italy; 131 Department of Physics and Astronomy, University of New Mexico, Albuquerque, NM United States of America; 132 Institute for Mathematics, Astrophysics and Particle Physics, Radboud University Nijmegen/Nikhef, Nijmegen, Netherlands; 133 Nikhef National Institute for Subatomic Physics and University of Amsterdam, Amsterdam, Netherlands; 134 Department of Physics, Northern Illinois University, DeKalb, IL United States of America; 135 Budker Institute of Nuclear Physics, SB RAS, Novosibirsk, Russia; 136 Department of Physics, New York University, New York, NY United States of America; 137 Ohio State University, Columbus, OH United States of America; 138 Faculty of Science, Okayama University, Okayama, Japan; 139 Homer L. Dodge Department of Physics and Astronomy, University of Oklahoma, Norman, OK United States of America; 140 Department of Physics, Oklahoma State University, Stillwater, OK United States of America; 141 RCPTM, Palacký University, Olomouc, Czech Republic; 142 Center for High Energy Physics, University of Oregon, Eugene, OR United States of America; 143 LAL, Université Paris-Sud and CNRS/IN2P3, Orsay, France; 144 Graduate School of Science, Osaka University, Osaka, Japan; 145 Department of Physics, University of Oslo, Oslo, Norway; 146 Department of Physics, Oxford University, Oxford, United Kingdom; 147 INFN Sezione di Pavia, Pavia, Italy; 148 Dipartimento di Fisica, Università di Pavia, Pavia, Italy; 149 Department of Physics, University of Pennsylvania, Philadelphia, PA United States of America; 150 Petersburg Nuclear Physics Institute, Gatchina, Russia; 151 INFN Sezione di Pisa, Pisa, Italy; 152 Dipartimento di Fisica E. Fermi, Università di Pisa, Pisa, Italy; 153 Department of Physics and Astronomy, University of Pittsburgh, Pittsburgh, PA United States of America; 154 Laboratorio de Instrumentacao e Fisica Experimental de Particulas - LIP, Lisboa, Portugal; 155 Departamento de Fisica Teorica y del Cosmos and CAFPE, Universidad de Granada, Granada, Spain; 156 Institute of Physics, Academy of Sciences of the Czech Republic, Praha, Czech Republic; 157 Czech Technical University in Prague, Praha, Czech Republic; 158 Faculty of Mathematics and Physics, Charles University in Prague, Praha, Czech Republic; 159 State Research Center Institute for High Energy Physics, Protvino, Russia; 160 Particle Physics Department, Rutherford Appleton Laboratory, Didcot, United Kingdom; 161 Physics Department, University of Regina, Regina, SK Canada; 162 Ritsumeikan University, Kusatsu, Shiga Japan; 163 INFN Sezione di Roma I, Roma, Italy; 164 Dipartimento di Fisica, Università La Sapienza, Roma, Italy; 165 INFN Sezione di Roma Tor Vergata, Roma, Italy; 166 Dipartimento di Fisica, Università di Roma Tor Vergata, Roma, Italy; 167 INFN Sezione di Roma Tre, Roma, Italy; 168 Dipartimento di Matematica e Fisica, Università Roma Tre, Roma, Italy; 169 Faculté des Sciences Ain Chock, Réseau Universitaire de Physique des Hautes Energies - Université Hassan II, Casablanca, Morocco; 170 Centre National de l’Energie des Sciences Techniques Nucleaires, Rabat, Morocco; 171 Faculté des Sciences Semlalia, Université Cadi Ayyad, LPHEA, Marrakech, Morocco; 172 Faculté des Sciences, Université Mohamed Premier and LPTPM, Oujda, Morocco; 173 Faculté des sciences, Université Mohammed V-Agdal, Rabat, Morocco; 174 DSM/IRFU (Institut de Recherches sur les Lois Fondamentales de l’Univers), CEA Saclay (Commissariat à l’Energie Atomique et aux Energies Alternatives), Gif-sur-Yvette, France; 175 Santa Cruz Institute for Particle Physics, University of California Santa Cruz, Santa Cruz, CA United States of America; 176 Department of Physics, University of Washington, Seattle, WA United States of America; 177 Department of Physics and Astronomy, University of Sheffield, Sheffield, United Kingdom; 178 Department of Physics, Shinshu University, Nagano, Japan; 179 Fachbereich Physik, Universität Siegen, Siegen, Germany; 180 Department of Physics, Simon Fraser University, Burnaby, BC Canada; 181 SLAC National Accelerator Laboratory, Stanford, CA United States of America; 182 Faculty of Mathematics, Physics & Informatics, Comenius University, Bratislava, Slovak Republic; 183 Department of Subnuclear Physics, Institute of Experimental Physics of the Slovak Academy of Sciences, Kosice, Slovak Republic; 184 Department of Physics, University of Johannesburg, Johannesburg, South Africa; 185 School of Physics, University of the Witwatersrand, Johannesburg, South Africa; 186 Department of Physics, Stockholm University, Stockholm, Sweden; 187 The Oskar Klein Centre, Stockholm, Sweden; 188 Physics Department, Royal Institute of Technology, Stockholm, Sweden; 189 Departments of Physics & Astronomy and Chemistry, Stony Brook University, Stony Brook, NY United States of America; 190 Department of Physics and Astronomy, University of Sussex, Brighton, United Kingdom; 191 School of Physics, University of Sydney, Sydney, Australia; 192 Institute of Physics, Academia Sinica, Taipei, Taiwan; 193 Department of Physics, Technion: Israel Institute of Technology, Haifa, Israel; 194 Raymond and Beverly Sackler School of Physics and Astronomy, Tel Aviv University, Tel Aviv, Israel; 195 Department of Physics, Aristotle University of Thessaloniki, Thessaloniki, Greece; 196 International Center for Elementary Particle Physics and Department of Physics, The University of Tokyo, Tokyo, Japan; 197 Graduate School of Science and Technology, Tokyo Metropolitan University, Tokyo, Japan; 198 Department of Physics, Tokyo Institute of Technology, Tokyo, Japan; 199 Department of Physics, University of Toronto, Toronto, ON Canada; 200 TRIUMF, Vancouver, BC Canada; 201 Department of Physics and Astronomy, York University, Toronto, ON Canada; 202 Faculty of Pure and Applied Sciences, University of Tsukuba, Tsukuba, Japan; 203 Department of Physics and Astronomy, Tufts University, Medford, MA United States of America; 204 Centro de Investigaciones, Universidad Antonio Narino, Bogota, Colombia; 205 Department of Physics and Astronomy, University of California Irvine, Irvine, CA United States of America; 206 INFN Gruppo Collegato di Udine, Udine, Italy; 207 ICTP, Trieste, Italy; 208 Dipartimento di Chimica, Fisica e Ambiente, Università di Udine, Udine, Italy; 209 Department of Physics, University of Illinois, Urbana, IL United States of America; 210 Department of Physics and Astronomy, University of Uppsala, Uppsala, Sweden; 211 Instituto de Física Corpuscular (IFIC) and Departamento de Física Atómica, Molecular y Nuclear and Departamento de Ingeniería Electrónica and Instituto de Microelectrónica de Barcelona (IMB-CNM), University of Valencia and CSIC, Valencia, Spain; 212 Department of Physics, University of British Columbia, Vancouver, BC Canada; 213 Department of Physics and Astronomy, University of Victoria, Victoria, BC Canada; 214 Department of Physics, University of Warwick, Coventry, United Kingdom; 215 Waseda University, Tokyo, Japan; 216 Department of Particle Physics, The Weizmann Institute of Science, Rehovot, Israel; 217 Department of Physics, University of Wisconsin, Madison, WI United States of America; 218 Fakultät für Physik und Astronomie, Julius-Maximilians-Universität, Würzburg, Germany; 219 Fachbereich C Physik, Bergische Universität Wuppertal, Wuppertal, Germany; 220 Department of Physics, Yale University, New Haven, CT United States of America; 221 Yerevan Physics Institute, Yerevan, Armenia; 222 Centre de Calcul de l’Institut National de Physique Nucléaire et de Physique des Particules (IN2P3), Villeurbanne, France

## Abstract

A measurement of splitting scales, as defined by the *k*_T_ clustering algorithm, is presented for final states containing a *W* boson produced in proton–proton collisions at a centre-of-mass energy of 7 TeV. The measurement is based on the full 2010 data sample corresponding to an integrated luminosity of 36 pb^−1^ which was collected using the ATLAS detector at the CERN Large Hadron Collider. Cluster splitting scales are measured in events containing *W* bosons decaying to electrons or muons. The measurement comprises the four hardest splitting scales in a *k*_T_ cluster sequence of the hadronic activity accompanying the *W* boson, and ratios of these splitting scales. Backgrounds such as multi-jet and top-quark-pair production are subtracted and the results are corrected for detector effects. Predictions from various Monte Carlo event generators at particle level are compared to the data. Overall, reasonable agreement is found with all generators, but larger deviations between the predictions and the data are evident in the soft regions of the splitting scales.

## Introduction

The CERN Large Hadron Collider (LHC), in addition to being a discovery machine, produces a wealth of data suitable for studies of the strong interaction. Due to the strongly interacting partons in the initial state and the large phase space available, final states often include hard jets arising from QCD bremsstrahlung. Discovery signals, on the other hand, often contain jets from quarks produced in electroweak interactions. A robust understanding of QCD-initiated processes in measurement and theory is necessary in order to distinguish such signals from backgrounds.

One critical background for searches is the *W*+jets process in the leptonic decay mode, which provides a large amount of missing transverse momentum together with jets and a lepton. This process is a testing ground for recent progress in QCD calculations, e.g. at fixed order [1, 2] or in combination with resummation [3–5], and it has been measured using many observables at both the Tevatron [6, 7] and the LHC [8–14].

In this paper the *k*_T_ jet finding algorithm [15, 16] is employed for a measurement of differential distributions of the *k*_T_ splitting scales in *W*+jets events. These measurements aim to provide results which can be interpreted particularly well in a theoretical context and improve the theoretical modelling of QCD effects. The measurement was performed independently in the electron (*W*→*eν*) and muon (*W*→*μν*) final states. Backgrounds such as multi-jet and top-quark pair production were subtracted and results were corrected for detector effects. The resulting data distributions are compared to predictions from various Monte Carlo event generators at particle level.

After an outline of the measurement in this section, the data analysis and event selection are summarised in Sect. 2. The Monte Carlo (MC) simulations used for theory comparisons are described in Sect. 3. Distributions at the detector level are displayed in Sect. 4. The procedure used to correct these to the particle level before any detector effects is outlined in Sect. 5 together with a weighting technique used to maximise the statistical power available, whilst minimising the systematic uncertainty arising from pileup. The evaluation of the systematic uncertainties is summarised in Sect. 6, and the results are shown in Sect. 7, followed by the conclusions in Sect. 8.

### Definition of *k*_T_ splitting scales

The *k*_T_ jet algorithm is a sequential recombination algorithm. Its splitting scales are determined by clustering objects together according to their distance from each other. The inclusive *k*_T_ algorithm uses the following distance definition [15, 16]: 1 where the transverse momentum *p*_T_, rapidity *y* and azimuthal angle *ϕ* of the input objects are labelled with an index corresponding to the *i*th and *j*th momentum in the input configuration, and *B* denotes a beam. These momenta can be determined using energy deposits in the calorimeter at the detector level, or hadrons at the particle level in Monte Carlo simulation. The *R* parameter was chosen to be *R*=0.6 in this paper, which is an intermediate choice between small values *R*≈0.2, whose narrow width minimizes the impact of pileup and the underlying event, and *R*≈1.0, whose large width efficiently collects radiation.

The clustering from the set of input momenta proceeds along the following lines:

1. Calculate *d*_*ij*_ and *d*_*iB*_ for all *i* and *j* from the input momenta according to Eq. (1).
2. Find their minimum:
  - If the minimum is a *d*_*ij*_, combine *i* and *j* into a single momentum in the list of input momenta: *p*_*ij*_=*p*_*i*_+*p*_*j*_
  - If the minimum is a *d*_*iB*_, remove *i* from the input momenta and declare it to be a jet.
3. Return to step 1 or stop when no particle remains.

The observables measured are defined as the smallest of the square roots of the *d*_*ij*_ and *d*_*iB*_ variables ($\sqrt{d_{ij}}$, $\sqrt{d_{iB}}$) found at each step in the clustering sequence. To simplify the notation they are commonly referred to as the splitting scales $\sqrt{d_{k}}$, which stand for the minima that occur when the input list proceeds from *k*+1 to *k* momenta by clustering and removing in each step. For example, $\sqrt{d_{0}}$ is found from the last step in the clustering sequence and reduces to the transverse momentum of the highest-*p*_T_ jet.

**Fig. 1:**
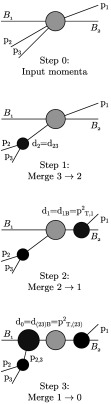
Illustration of the *k*_T_ clustering sequence starting from the original input configuration (three objects *p*_1_, *p*_2_, *p*_3_, and beams *B*_1_, *B*_2_). At each step, *k*+1 objects are merged to *k*

Figure 1 schematically displays the clustering sequence derived from an original input configuration of three objects labelled *p*_1_, *p*_2_, *p*_3_ in the presence of beams *B*_1_ and *B*_2_. In the first clustering step, where three objects are grouped into two (denoted 3→2), the minimal splitting scale is found between momenta *p*_2_ and *p*_3_, leading to *d*_2_=*d*_23_. In the second step (2→1), the momentum *p*_1_ is closest to the beam, and thus is removed and declared a jet at the scale $d_{1}=d_{1B}=p_{\mathrm{T}1}^{2}$. Ultimately, the third clustering (1→0) has only the beam distance of the combined input *p*_2,3_ remaining, leading to a scale of $d_{0}=d_{(23)B}=p_{\mathrm{T},(23)}^{2}$.

### Features of the observables

An important feature of these observables is their separation into two regions: a “hard” one with $\sqrt{d_{k}}\gtrsim20~\mathrm{GeV}$ which is dominated by perturbative QCD effects, and a “soft” one in which more phenomenological modelling aspects such as hadronisation and multiple partonic interactions may exert substantial influence on theory predictions. The number of events in the hard region for high *k* is naturally low in the data sample analysed for this measurement. Thus for statistical reasons values of 0≤*k*≤3 are considered in this publication. No explicit jet requirement is imposed in the event selection.

In addition to the observables mentioned above, it is also interesting to study ratios of consecutive clustering values, $\sqrt{d_{k+1}/d_{k}}$, where some experimental uncertainty cancellations occur, as discussed in Sect. 6. Of particular interest is the region where $\sqrt{d_{k+1}/d_{k}}\to 1$, as it probes events with subsequent emissions at similar scales. Those events could be challenging to describe correctly for parton shower generators without matrix element corrections. The splitting scale ratio amounts to a normalisation of the splitting scale to the scale of the QCD activity in the “underlying process”, i.e. after the clustering. To reduce the influence of non-perturbative effects, each ratio observable $\sqrt{d_{k+1}/d_{k}}$ is measured with events satisfying $\sqrt {d_{k}}>20~\mathrm{GeV}$.

The central idea underlying this measurement is that the measure of the *k*_T_ algorithm corresponds relatively well to the singularity structure of QCD. To illustrate this, the small-angle limit of the squared *k*_T_ measure is given in terms of the angle *θ*_*ij*_ between two momenta *i* and *j*, and the energy corresponding to the softer momentum, *E*_*i*_, by Ref. [15]: 2
3 while the splitting probability for a final-state branching into partons *i* and *j* evaluates to

4 $$ \frac{\mathrm{d}P_{ij\to i,j}}{\mathrm{d}E_i\mathrm{d}\theta_{ij}} \sim\frac{1}{\min(E_i,E_j)\theta_{ij}} $$

in the collinear limit [17].

From a comparison of Eqs. (2) and (4) it can be seen that each step of the *k*_T_ algorithm identifies the parton pair which would be the most likely to have been produced by QCD interactions. In that sense, this clustering sequence mimicks the reversal of the QCD evolution.

In contrast the anti-*k*_*t*_ [18] algorithm cannot be used in the same way: its distance measure replaces all $p_{\mathrm{T}}^{2}$ by $p_{\mathrm{T}}^{-2}$. So even though collinear branchings are still clustered first, the same is not true for soft emissions anymore. Thus the splitting structure within the anti-*k*_*t*_ algorithm must be constructed via the *k*_T_ splitting algorithm [19].

Just like QCD matrix elements, the *k*_T_ splitting scales provide a unified view of initial- and final-state radiation. Through the combination of the distance to the beams and the relative distance of objects to each other, the $\sqrt{d_{k}}$ distributions contain information about both the *p*_T_ spectra and the substructure of jets.

### Existing predictions and measurements

The *k*_T_ splittings and related distributions have attracted the attention of theorists, in *W*→*ℓν* and similar final states. They can be resummed analytically at next-to-leading-logarithm accuracy as demonstrated for the example of jet production by QCD processes in hadron collisions in Refs. [20, 21]. The ratio observable *y*_23_ defined by the authors is closely related to the ratio observables $\sqrt{d_{k+1}/d_{k}}$ in this analysis. Other theoretical studies may be found in Refs. [22, 23].

Experimentally, these kinds of observables were measured at LEP [24–26] using the *e* ^+^ *e* ^−^ (Durham) *k*_T_ algorithm. Their theoretical features (resummability) were used in Refs. [27, 28] to determine *α*_*s*_ with high precision. Related observables were also measured at HERA [29–32].

## Data analysis

### The ATLAS detector

The ATLAS detector [33] at the LHC covers nearly the entire solid angle around the collision point. It consists of an inner tracking detector surrounded by a thin superconducting solenoid, electromagnetic and hadronic calorimeters, and a muon spectrometer incorporating three large superconducting toroid magnets.

The inner-detector system is immersed in a 2 T axial magnetic field and provides charged particle tracking in the range |*η*|<2.5.^1^ The high-granularity silicon pixel detector covers the vertex region and typically provides three measurements per track. It is followed by the silicon microstrip tracker which usually provides four two-dimensional measurement points per track. These silicon detectors are complemented by the transition radiation tracker, which contributes to track reconstruction up to |*η*|=2.0. The transition radiation tracker also provides electron identification information based on the fraction of hits (typically 30 in total) above a higher energy-deposit threshold corresponding to transition radiation.

The calorimeter system covers the pseudorapidity range |*η*|<4.9. Within the region |*η*|<3.2, electromagnetic calorimetry is provided by barrel and endcap high-granularity lead/liquid-argon (LAr) calorimeters, with an additional thin LAr presampler covering |*η*|<1.8 to correct for energy loss in material upstream of the calorimeter. Hadronic calorimetry is provided by a steel/scintillator-tile calorimeter, segmented radially into three barrel structures within |*η*|<1.7, and two copper/LAr hadronic endcap calorimeters. The solid angle coverage is completed with forward copper/LAr and tungsten/LAr calorimeter modules optimised for electromagnetic and hadronic measurements respectively.

The muon spectrometer comprises separate trigger and high-precision tracking chambers measuring the deflection of muons in a magnetic field generated by superconducting air-core toroids. The precision chamber system covers the region |*η*|<2.7 with three layers of monitored drift tubes, complemented by cathode strip chambers in the forward region, where the background is highest. The muon trigger system covers the range |*η*|<2.4 with resistive plate chambers in the barrel, and thin gap chambers in the endcap regions.

A three-level trigger system is used to select interesting events [34]. The Level-1 trigger is implemented in hardware and uses a subset of detector information to reduce the event rate to a design value of at most 75 kHz. This is followed by two software-based trigger levels which together reduce the event rate to about 200 Hz.

### Event selection

The selection of *W* events is based on the criteria described in Refs. [13, 35] and summarised briefly below.

#### Data sample and trigger

The entire 2010 data sample at $\sqrt{s}=7~\mathrm{TeV}$ was used, corresponding to an integrated luminosity of approximately 36 pb^−1^. The 2010 data sample was chosen due to the low pileup conditions during data taking, where the mean number of interactions per bunch crossing was at most 2.3 during that period. In the *W*→*μν* analysis, the first few pb^−1^ were excluded to restrict to a data sample of events recorded with a uniform trigger configuration and optimal detector performance.

Single-lepton triggers were used to retain *W*→*ℓν* candidate events. For the electron channel a trigger threshold of 14 GeV for early data-taking periods and 15 GeV for later data-taking periods was applied. For the muon channel a trigger threshold of 13 GeV was applied. All relevant detector components were required to be fully operational during the data taking. Events with at least one reconstructed interaction vertex within 200 mm of the interaction point in the *z* direction and having at least three associated tracks were considered. The number of reconstructed vertices reflects the pileup conditions and, in both channels, was used to reweight the MC simulation to improve its modelling of the pileup conditions observed in data. The number of reconstructed vertices was also used to estimate the uncertainty due to possible mismodelling of the pileup.

#### Electron selection

Clusters formed from energy depositions in the electromagnetic calorimeter were required to have matched tracks, with the further requirement that the cluster shapes are consistent with electromagnetic showers initiated by electrons. On top of the tight identification criteria, a calorimeter-based isolation requirement for the electron was applied to further reduce the multi-jet background. Additional requirements were applied to remove electrons falling into calorimeter regions with non-operational LAr readout. The kinematic requirements on the electron candidates included a transverse momentum requirement $p_{\mathrm{T}}^{\ell}>20~\mathrm{GeV}$ and pseudorapidity |*η* ^*ℓ*^|<2.47 with removal of the transition region 1.37<|*η* ^*ℓ*^|<1.52 between the calorimeter modules. Exactly one of these selected electrons was required for the *W*→*eν* selection. In constructing the *k*_T_ cluster sequence, clusters of calorimeter cells included in a reconstructed jet within Δ*R*=0.3 of the electron candidate were removed from the input configuration.

#### Muon selection

Muon candidates were required to have tracks reconstructed in both the muon spectrometer and inner detector, with $p_{\mathrm{T}}^{\ell}$ above 20 GeV and pseudorapidity |*η* ^*ℓ*^|<2.4. Requirements on the number of hits used to reconstruct the track in the inner detector were applied, and the muon’s point of closest approach to the primary vertex was required to be displaced in *z* by less than 10 mm. Track-based isolation requirements were also imposed on the reconstructed muon. At least one muon was required for the *W*→*μν* selection. To retain consistency with the acceptance in the electron channel, when constructing the *k*_T_ cluster sequence, clusters of calorimeter cells falling close to the muon candidate were removed from the input configuration as in the electron selection.

#### Selection of *W* candidate events and construction of observables

The *W*→*ℓν* event selection required that the magnitude of the missing transverse momentum, $E_{\mathrm{T}}^{\mathrm{miss}}$ [36], be greater than 25 GeV. The reconstructed transverse mass obtained from the lepton transverse momentum $\vec{p}_{\mathrm{T}}^{\ell}$ and $\vec{E}_{\mathrm {T}}^{\mathrm{miss}}$ vectors was required to fulfill $m_{\mathrm {T}}^{W}=\sqrt{2(p_{\mathrm{T}}^{\ell}E_{\mathrm{T}}^{\mathrm{miss}}-\vec{p}_{\mathrm{T}}^{\ell }\cdot\vec{E}_{\mathrm{T}}^{\mathrm{miss}})}>40~\mathrm{GeV}$. No requirements were made with respect to the number of reconstructed jets in the event.

The observables defined in Sect. 1.1 were constructed using calorimeter energy clusters within a pseudorapidity range of |*η* ^cl^|<4.9. The clusters were seeded by calorimeter cells with energies at least 4*σ* above the noise level. The seeds were then iteratively extended by including all neighbouring cells with energies at least 2*σ* above the noise level. The cell clustering was finalised by the inclusion of the outer perimeter cells around the cluster. The so-called topological clusters that resulted were calibrated to the hadronic energy scale [37, 38], by applying weights to account for calorimeter non-compensation, energy lost upstream of the calorimeters and noise threshold effects.

### Background treatment

The contributions of electroweak backgrounds (*Z*→*ℓℓ*, *W*→*τν* and diboson production), as well as $t\overline{t}$ and single-top-quark production, to both channels were estimated using the MC simulation. The absolute normalisation was derived using the total theoretical cross sections and corrected using the acceptance and efficiency losses of the event selection. The shape and normalisation of the distributions of various observables for the multi-jet background were determined using data-driven methods in both analysis channels. For the *W*→*eν* selection, the background shape was obtained from data by reversing certain calorimeter-based electron identification criteria to produce a multi-jet-enriched sample. Similarly, to estimate the multi-jet contribution to *W*→*μν*, the background shape was obtained from data by inverting the requirements on the muon transverse impact parameter and its significance. These multi-jet enriched samples provided the shapes of the distributions of multi-jet background observables. The normalisation of the multi-jet background was determined by fitting a linear combination of the multi-jet and leptonic $E_{\mathrm{T}}^{\mathrm{miss}}$ shapes to the observed $E_{\mathrm{T}}^{\mathrm{miss}}$ distribution, following the procedures described in Refs. [13, 35]. The total background was thus estimated to be 5 % of the signal for the *W*→*eν* analysis, with the largest contribution arising from multi-jet production. For the *W*→*μν* analysis, the total background is 9 % of the signal and is dominated by the *Z*→*ℓℓ* process. At large splitting scales, top quark pair production becomes the dominant contribution in both channels.

## Monte Carlo simulations

All detector-level studies and the extraction of particle-level distributions involved two signal MC generators, Alpgen + Herwig and Sherpa. Alpgen v2.13 [39], a matrix-element (ME) generator, was interfaced to Herwig v6.510 [40] for parton showering (PS) and hadronisation, and to Jimmy v4.31 [41] for multiple parton interactions. The MLM [22] matching scheme was used to combine *W*-boson production samples having up to five partons with the parton shower, with the matching scale set at 20 GeV. Sherpa v1.3.1 [42] was used to generate an alternative signal sample of events with *W*+jets, using a ME+PS merging approach [23] to prevent double counting from the parton shower, and extending the original CKKW method [43] by taking into account truncated shower emissions. Up to five partons were generated in the ME and the matching scale was set to 30 GeV.

The single-top-quark background events were generated at next-to-leading-order (NLO) accuracy using the Mc@Nlo v3.3.1 [44] generator. Mc@Nlo was interfaced to Herwig and Jimmy. The Powheg v1.01 [45] generator, interfaced to Pythia6 v6.421 [46], was used to simulate the $t\bar{t}$ background. The background from diboson production was generated using Herwig. Backgrounds from inclusive *Z* production were simulated using Pythia6.

Three sets of parton density functions (PDFs) were used in these MC samples: CTEQ6L1 [47] for the Alpgen samples and the parton showering and underlying event in the Powheg samples interfaced to Pythia6; MRST 2007 LO^∗^ [48] for Pythia6 and Herwig; and CTEQ6.6M [49] for Mc@Nlo, Sherpa, and the NLO matrix element calculations in Powheg. The underlying event tunes were AUET1 [50] for the Herwig, Alpgen, and Mc@Nlo samples, and AMBT1 [51] for the Pythia6 and Powheg samples. The samples generated with Sherpa used the default underlying event tune.

Each generated event was passed through the standard ATLAS detector simulation [52], based on Geant4 [53]. The MC events were reconstructed and analysed using the same software chain as applied to the data. The resulting MC predictions for the samples were normalised to their respective theoretical cross sections calculated at NLO [13], with the exception of the *W* and *Z* samples which were normalised to NNLO [54], and the multi-jet background which was normalised to a value extracted from the data as is described in Sect. 2.

At the particle level, some additional *W*+jets NLO MC generators were compared to the final results. The Powheg [45, 55] samples were matched to Pythia6 v6.425 or Pythia8 v8.165 [56] for parton showering and hadronisation, while another sample was generated with Mc@Nlo v4.06 [44] using Herwig v6.520.2. The Sherpa Menlops sample used Sherpa v1.4.1 with its built-in Menlops method [4], allowing an NLO+PS matched sample for inclusive *W* production [57] to be merged with LO matrix elements for a *W* boson and up to five partons using a matching scale at 20 GeV. All these NLO samples were generated with the CT10 PDF set [58].

The Mc@Nlo, Powheg and Alpgen + Herwig samples were supplemented with a simulation of QED final-state radiation using Photos v2.15.4 [59] and tau decays using Tauola v27feb06 [60]. The Sherpa samples included QED final-state radiation in a different resummation approach [61] and a built-in tau decay algorithm.

## Detector-level comparisons of Monte Carlo to data

**Fig. 2:**
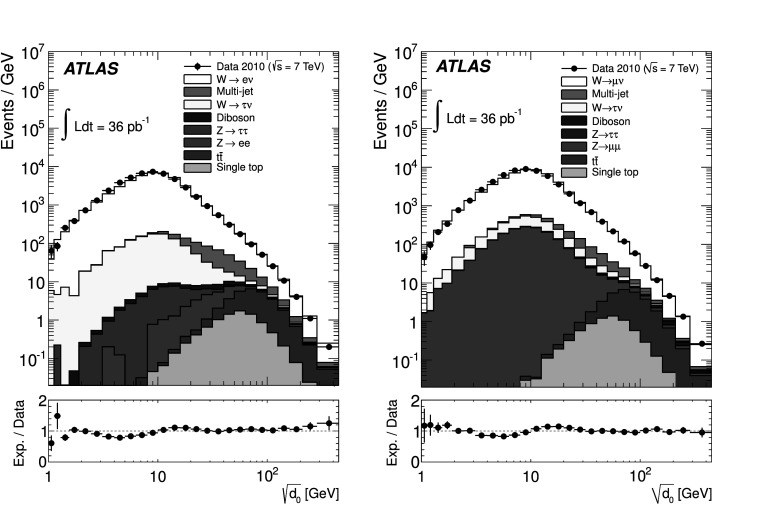
Uncorrected splitting scale $\sqrt{d_{0}}$ for events passing the *W*→*eν* (*left*) and *W*→*μν* (*right*) selection requirements. The distributions from the data (markers) are compared with the predicted signal from the MC simulation, provided by Alpgen + Herwig and normalised to the NNLO prediction. In addition, physics backgrounds, also shown, have been added in proportion to the predictions from the MC simulation. The ratio between the expectation and the data is shown in the lower plot. The error bars shown on the data are statistical only

**Fig. 3:**
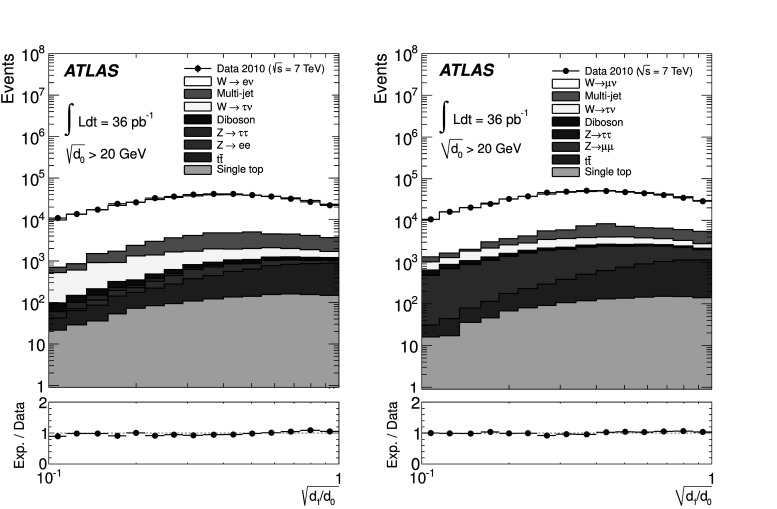
Uncorrected ratio $\sqrt{d_{1}/d_{0}}$ for events passing the *W*→*eν* (*left*) and *W*→*μν* (*right*) selection requirements. The distributions from the data (markers) are compared with the predicted signal from the MC simulation, provided by Alpgen + Herwig and normalised to the NNLO prediction. In addition, physics backgrounds, also shown, have been added in proportion to the predictions from the MC simulation. The ratio between the expectation and the data is shown in the *lower plot*. The error bars shown on the data are statistical only

The observed and expected detector-level distributions for $\sqrt {d_{0}}$ in the electron and muon channels are shown in Fig. 2, where the MC signal predictions are provided by Alpgen + Herwig normalised to NNLO predictions [54]. The *W*-boson kinematic distributions are shown in detail in Refs. [13, 35]. The corresponding plots for $\sqrt{d_{1}}$, $\sqrt{d_{2}}$ and $\sqrt {d_{3}}$ can be found Figs. 9, 10 and 11 in Appendix A.1. Figure 3 shows the ratio of the second-hardest to the hardest splitting scale in each event. Again, the sub-leading ratio distributions at detector level are displayed in Appendix A.1. For the hardest clustering in the event, $\sqrt{d_{0}}$, generally good agreement between the Alpgen + Herwig MC predictions and the data is observed. The agreement is similar for both the electron and the muon channels.

## Particle-level extraction

### Corrections for detector effects

After subtraction of backgrounds, the detector level distributions were corrected (“unfolded”) to the final-state particle level separately for the two channels, taking into account the effects of pileup and detector response. The unfolding was performed with the RooUnfold [62] package, using a Bayesian algorithm[63], in which Bayes theorem was used to derive the particle-level distributions from the detector-level distributions, over three iterations. The input for the algorithm at particle and detector level was taken from the Alpgen + Herwig sample as a default. Both the MC simulation and data-driven methods were used to demonstrate that this iterative Bayesian method was able to recover the corresponding particle-level distributions.

The selection requirements applied to the event at the particle level are:

- $p_{\mathrm{T}}^{\ell}> 20~\mathrm{GeV}$ (*ℓ*=electron *e* or muon *μ*)
- |*η* ^*e*^|<2.47 excluding 1.37<|*η* ^*e*^|<1.52
- |*η* ^*μ*^|<2.4
- $p_{\mathrm{T},\mathrm{lead}}^{\nu} > 25~\mathrm{GeV}$ (*ν*_lead_=highest-*p*_T_ neutrino in event)
- $m_{\mathrm{T}}^{W} > 40~\mathrm{GeV}$

Only events with exactly one lepton passing the requirements were taken into account. Leptons were defined to include all photon radiation within a cone of Δ*R*=0.1 around the final-state lepton as suggested in Ref. [64]. All lepton requirements were calculated from these combined objects. The observables defined in Sect. 1.1 were constructed using all stable particles within a pseudorapidity range of |*η* ^cl^|<4.9 with lifetime greater than 10 ps, excluding the lepton and neutrino originating from the *W* boson decay.

### Weighted combination

To reduce the impact of imperfect MC modelling of pileup effects, whilst optimising the statistical power available, two different event samples were defined and utilised as follows.

- “Low-pileup sample”: exactly one reconstructed vertex was required in data. The response matrices used to unfold the data and the background templates were also constructed from events where exactly one reconstructed vertex was required.
- “High-pileup sample”: as above, with the difference that the number of reconstructed vertices was required to be greater than one.

At large $\sqrt{d_{k}}$, the statistical uncertainty of the high-pileup sample is smaller than that in the low-pileup sample. However, at small $\sqrt{d_{k}}$, the systematic pileup uncertainty of the low-pileup sample is smaller than that in the high-pileup sample. To minimise the overall uncertainty on the measurement, the distributions were combined as follows. For each bin of the final distribution, the best estimate *N* was calculated from the bin contents *N*_1_, *N*_2_ of the distributions in the low-pileup and high-pileup samples respectively, as

5 $$ N=\frac{N_1\cdot{W_1} + N_2\cdot{W_2}}{{W_1}+{W_2}}. $$

The weights *W*_*i*_ for each sample were constructed from the inverse of the sum in quadrature of the statistical and pileup uncertainties on the low-pileup and the high-pileup samples. The evaluation of the pileup uncertainty on each sample is described in detail in Sect. 6. The statistical uncertainty of the final distribution was calculated assuming no correlation between the two samples.

## Systematic uncertainties

To evaluate the impact of a particular source of systematic uncertainty at the particle level, the observable considered was varied within its uncertainty, the response matrix was recalculated taking this variation into account, and the new response matrix was used to unfold the data. The fractional shift in the resulting unfolded data from nominal was interpreted as the systematic uncertainty due to that particular effect. The separate sources of uncertainty are described in the following.

The relative systematic uncertainty on the energy scale of the topological clusters was evaluated from a combination of MC studies and single-pion response measurements [36] to be $1 \pm a \times (1 + b / p_{\mathrm{T}}^{\mathrm{cl}})$ where $p_{\mathrm{T}}^{\mathrm{cl}}$ represents the transverse momentum of each cluster. The constants *a* and *b* were determined to be *a*=3 (10) % when |*η* ^cl^|<3.2 (|*η* ^cl^|>3.2), and *b*=1.2 GeV. A shift of the cluster energy results in a shift of the distributions to higher or lower values. The uncertainty due to the cluster energy scale was thus evaluated separately for the low-pileup and high-pileup distributions and combined in a weighted linear sum. The uncertainty ranges from 5 % to 55 % for the splitting scales $\sqrt{d_{k}}$ and from 2 % to 85 % for the $\sqrt{d_{k+1}/d_{k}}$ ratio distributions.

The lepton trigger, identification and reconstruction efficiencies as well as the lepton energy scale and resolution were measured in data using *Z*→*ℓℓ* events via the tag-and-probe method, as described in Refs. [13, 35, 65]. The uncertainty is less than 3 % for the splitting scales $\sqrt{d_{k}}$ and less than 1 % for the $\sqrt{d_{k+1}/d_{k}}$ ratio distributions.

The systematic uncertainty due to possible MC mismodelling of pileup was evaluated separately on the low-pileup and high-pileup distributions. The impact of pileup mismodelling on the low-pileup sample was evaluated by varying the requirements on the *z*-displacement of the interaction vertex and the number of associated tracks. An additional uncertainty accounts for the possible mismodelling of contributions from adjacent bunch-crossings. It was evaluated by comparing two different data-taking periods: one in which proton bunches were arranged in trains, and the other without bunch trains. The impact of pileup mismodelling on the high-pileup sample was evaluated as the fractional difference between the particle-level measurements for the low-pileup and the high-pileup events, with the statistical uncertainty subtracted in quadrature. The uncertainty ranges from 1 % to 30 % for the splitting scales $\sqrt{d_{k}}$ and is largest for small splitting scales. For the $\sqrt {d_{k+1}/d_{k}}$ ratio distributions the uncertainty ranges from 1 % to 15 %.

The uncertainty inherent in the unfolding procedure itself was estimated by reweighting the response matrix in the unfolding such that Alpgen + Herwig would accurately model the distribution under consideration as measured from data at reconstruction level. A second variation was performed by creating a response matrix from Sherpa. The larger effect, per bin, obtained from these two estimates of the systematic uncertainty was taken as the systematic uncertainty due to unfolding. The uncertainty ranges between 5 % and 55 % for the splitting scales $\sqrt{d_{k}}$, being largest for small values of $\sqrt{d_{k}}$ and in the vicinity of $\sqrt{d_{k}} \approx15~\mathrm{GeV}$. For the $\sqrt{d_{k+1}/d_{k}}$ ratio distributions the uncertainty ranges between 1 % and 35 %.

The systematic uncertainties on the electroweak and top-quark background normalisations were assigned using the theoretical uncertainty on the cross section of each process under consideration. The uncertainty on the multi-jet background normalisation was obtained by varying the methods used for extracting this value from data, as described in Refs. [13, 35]. An additional uncertainty was included on the shape of the multi-jet contribution, which was derived by comparing data-driven and simulation estimates of this background contribution. The uncertainty ranges from 0.5 % to 15 % for the splitting scales $\sqrt{d_{k}}$ and from 1 % to 20 % for the $\sqrt{d_{k+1}/d_{k}}$ ratio distributions.

**Fig. 4:**
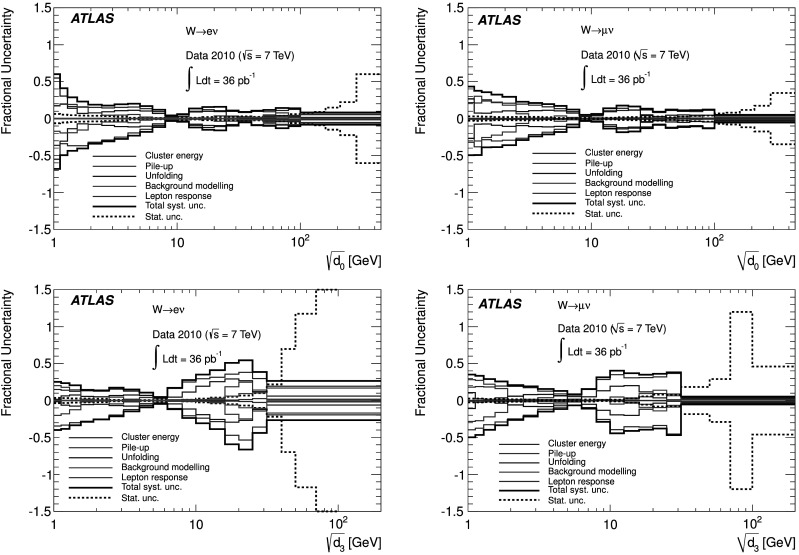
Summary of the systematic uncertainties on the measured particle-level distributions for $\sqrt{d_{0}}$ (*top*) and $\sqrt {d_{3}}$ (*bottom*) in the *W*→*eν* (*left*) and *W*→*μν* (*right*) channels

**Fig. 5:**
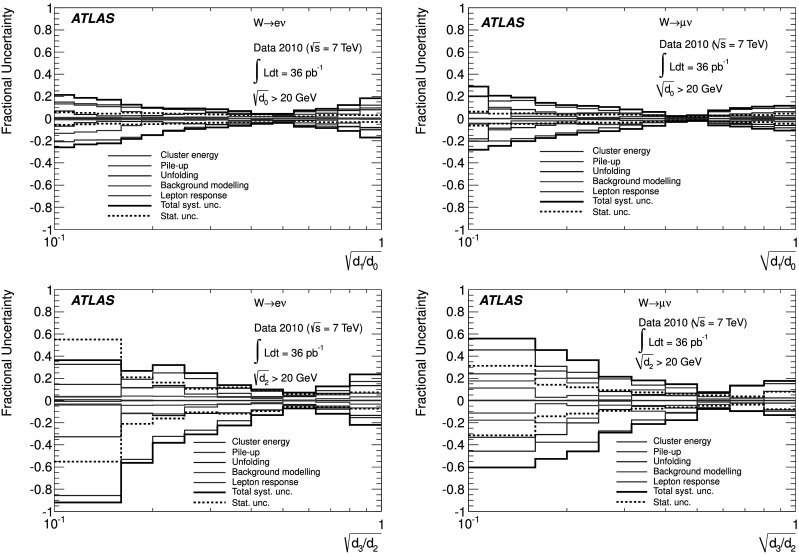
Summary of the systematic uncertainties on the measured particle-level ratios for $\sqrt{d_{1}/d_{0}}$ (*top*) and $\sqrt {d_{3}/d_{2}}$ (*bottom*) in the *W*→*eν* (*left*) and *W*→*μν* (*right*) channels

The magnitudes of the separate uncertainties for the hardest and fourth-hardest splittings are summarised in Figs. 4 and 5, where the statistical errors are also shown. Other cases are available in Appendix A.2. The cluster energy scale, pileup, and the unfolding procedure are the dominant sources of uncertainty in both the electron and muon channels.

For each uncertainty an error band was calculated, where the upper limit is defined as the variation leading to larger values compared to the nominal distribution and the lower limit as the variation leading to lower values. To avoid underestimating the uncertainty in bins where statistical fluctuations were large, if both variations led to a shift in the same direction the larger difference with respect to the nominal distribution was taken as a symmetric uncertainty. Correlations between separate sources of systematic uncertainties and between different bins of the distributions were not considered. The quadratic sum of all systematic uncertainties considered above was taken to be the overall systematic uncertainty on the distributions. The overall systematic uncertainty ranges between 10 % and 60 % for the $\sqrt{d_{k}}$ distributions, being largest for small splitting scales and in the vicinity of $\sqrt{d_{k}} \approx15~\mathrm{GeV}$. The uncertainty is smallest in the vicinity of $\sqrt{d_{k}} \approx10~\mathrm{GeV}$ as this corresponds to the peak of the distribution and is thus less sensitive to scale uncertainties. For the $\sqrt{d_{k+1}/d_{k}}$ ratio distributions the overall systematic uncertainty ranges between 5 % and 95 %, being largest for small values of the ratios. The statistical uncertainty on the unfolded measurement was combined in quadrature with the systematic uncertainty to obtain the total uncertainty.

## Results

**Fig. 6:**
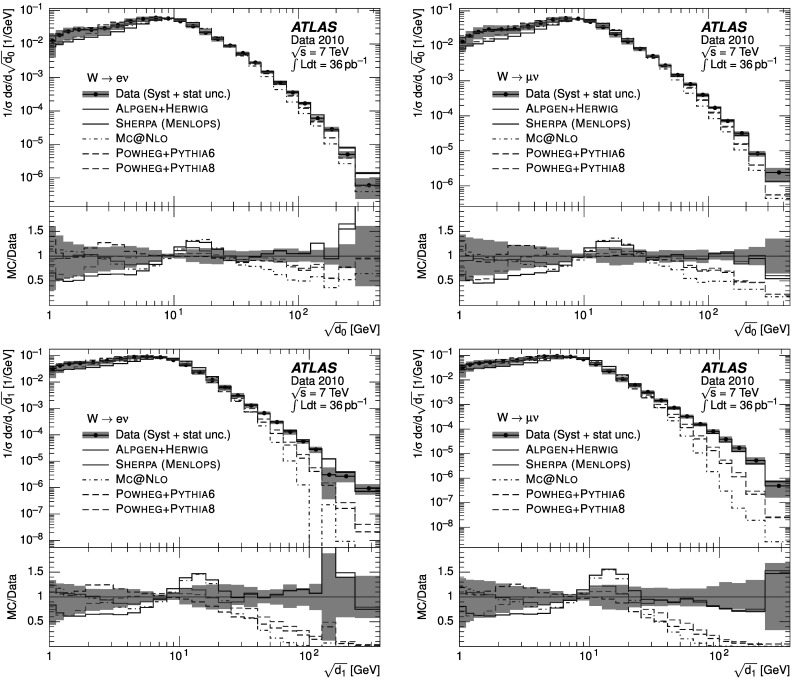
Distributions of $\sqrt{d_{0}}$ (*top*) and $\sqrt{d_{1}}$ (*bottom*) in the *W*→*eν* (*left*) and *W*→*μν* (*right*) channels, shown at particle level. The data (markers) are compared to the predictions from various MC generators, and the *shaded bands* represent the quadrature sum of systematic and statistical uncertainties on each bin. The histograms have been normalised to unity

**Fig. 7:**
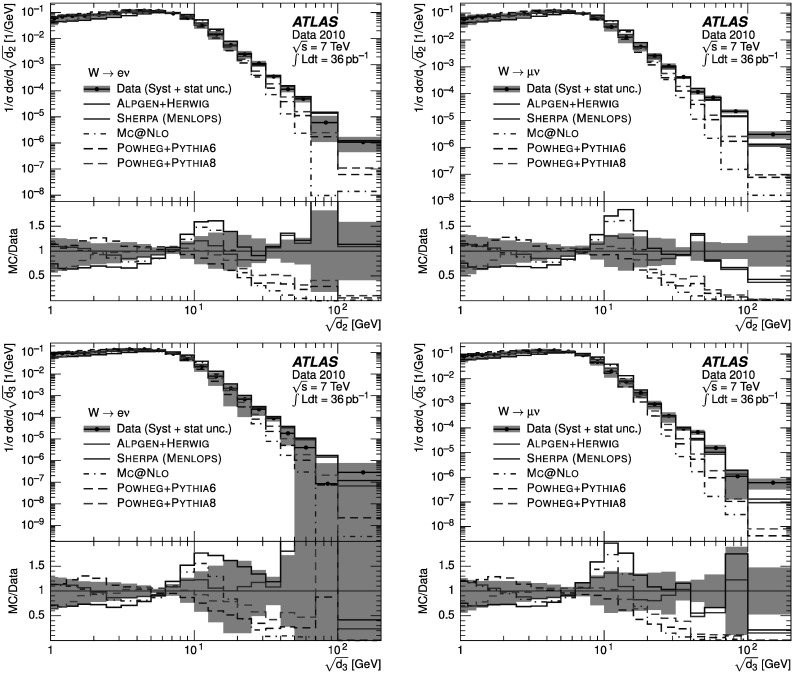
Distributions of $\sqrt{d_{2}}$ (*top*) and $\sqrt{d_{3}}$ (*bottom*) in the *W*→*eν* (*left*) and *W*→*μν* (*right*) channels, shown at particle level. The data (markers) are compared to the predictions from various MC generators, and the *shaded bands* represent the quadrature sum of systematic and statistical uncertainties on each bin. The histograms have been normalised to unity

The different MC simulations in Sect. 3 were compared to the data using Rivet [66]. The FastJet library [19] was used to construct the *k*_T_ cluster sequence. Figures 6 and 7 display the $\sqrt{d_{k}}$ distributions, which have been individually normalised to unity to allow for shape comparisons.

The Alpgen + Herwig MC simulation generally agrees very well with the data, as already seen in the detector-level distributions. The discrepancies between the MC and data distributions are covered by the systematic and statistical uncertainties. The Sherpa predictions are almost identical to those from Alpgen + Herwig in the hard region of the distributions, $\sqrt {d_{k}}>20~\mathrm{GeV}$, where tree-level matrix elements are applied.

All three generators based on NLO+PS methods, i.e. Mc@Nlo, Powheg + Pythia6 and Powheg+Pythia8, predict significantly less hard activity than that found in data. As expected, this effect is strongest for higher multiplicities *k*≥1, where in NLO+PS generators no matrix elements are used for the description of the QCD emission. It is interesting that they also do not describe well the hard tail of the hardest splitting scale $\sqrt{d_{0}}$, even though they are nominally at the same leading-order accuracy as Alpgen+Herwig and Sherpa in this distribution. This may be due to differences in higher-multiplicity parton processes becoming relevant in that region or different scale choices in the real-emission matrix element or a combination of both.

In the intermediate region of 10–20 GeV, both Sherpa and Mc@Nlo show a similar excess over data in all $\sqrt{d_{k}}$. For Sherpa it is compensated by an undershoot in the very soft region, while for Mc@Nlo the soft region is described well. Powheg + Pythia6 and Powheg + Pythia8 also agree with data in the soft region, and their deviations from each other due to the differences in parton showering and hadronisation lie within the experimental uncertainties. They give identical predictions for the hard region of $\sqrt{d_{0}}$, where both of them should be dominated by an identical real-emission matrix element. This confirms the expectation that the hard region is dominated by perturbative effects while resummation and non-perturbative effects have a large influence in the softer regions.

**Fig. 8:**
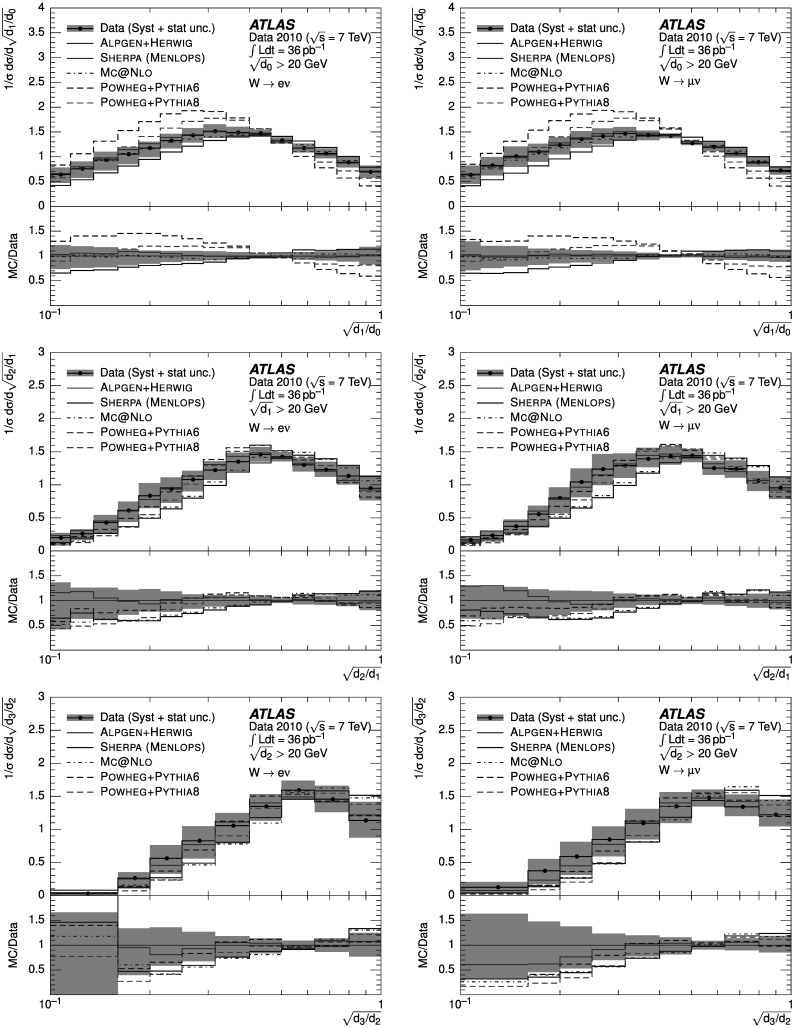
Distributions of the $\sqrt {d_{k+1}/d_{k}}$ ratio distributions for *W*→*eν* (*left*) and *W*→*μν* (*right*) in the data after correcting to particle level (marker) in comparison with various MC generators as described in the text. The *shaded bands* represent the quadrature sum of systematic and statistical uncertainties on each bin. The histograms have been normalised to unity

**Fig. 9:**
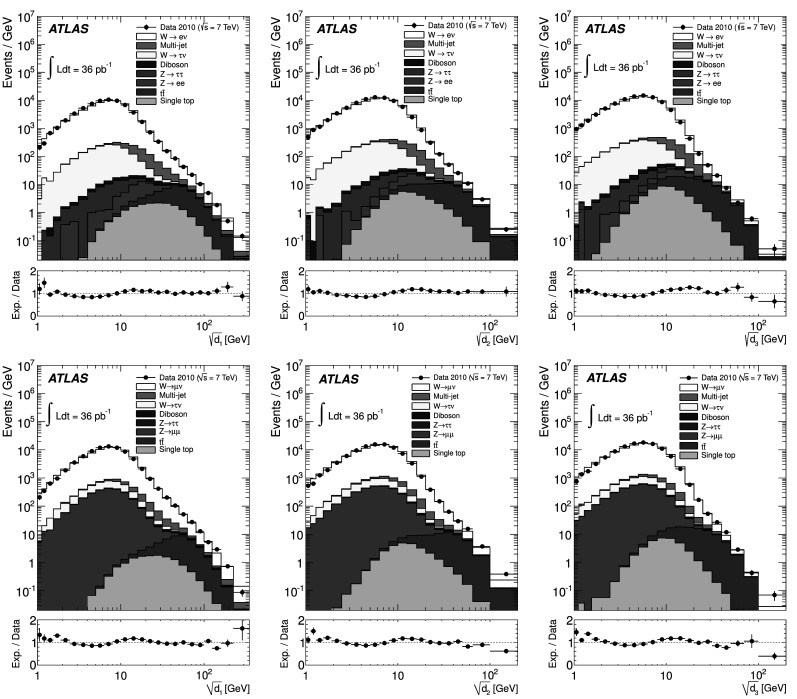
Uncorrected splitting scales $\sqrt{d_{1}}$ (*left*), $\sqrt {d_{2}}$ (*middle*) and $\sqrt{d_{3}}$ (*right*) for events passing the *W*→*eν* (*top*) and *W*→*μν* (*bottom*) selection requirements. The distributions from the data (markers) are compared with the predicted signal from the MC simulation, provided by Alpgen + Herwig and normalised to the NNLO prediction. In addition, physics backgrounds, also shown, have been added in proportion to the predictions from the MC simulation. The ratio between the expectation and the data is shown in the *lower plot*. The error bars shown on the data are statistical only

**Fig. 10:**
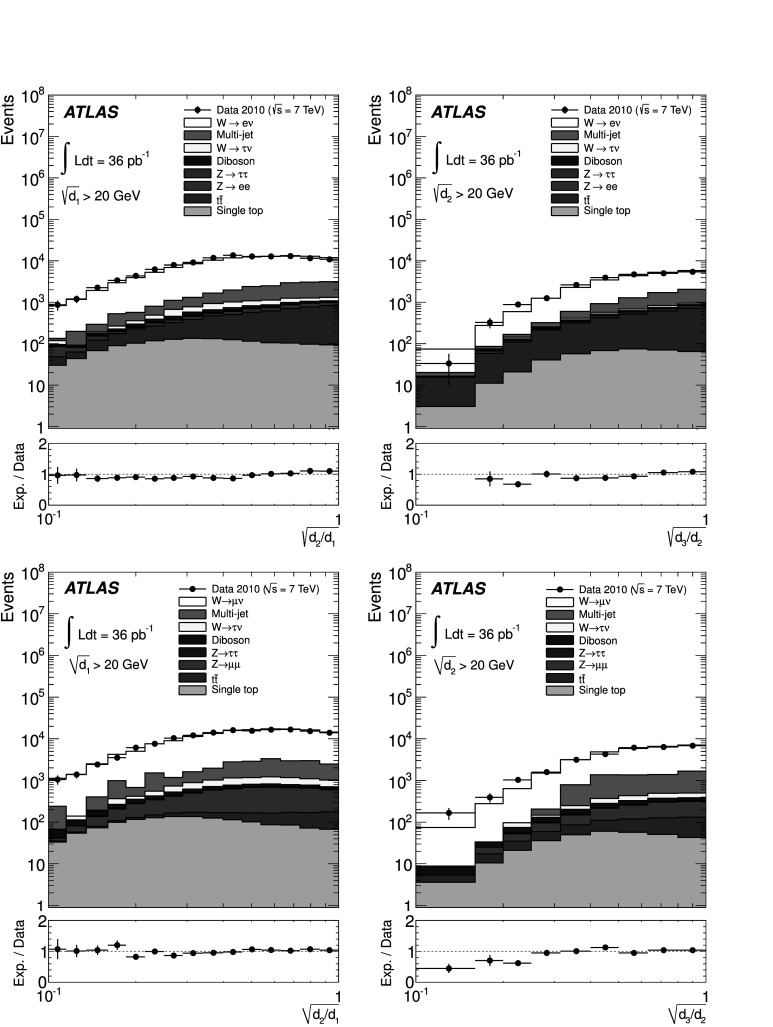
Uncorrected ratios $\sqrt{d_{2}/d_{1}}$ (*left*) and $\sqrt {d_{3}/d_{2}}$ (*right*) for events passing the *W*→*eν* (*top*) and *W*→*μν* (*bottom*) selection requirements. The distributions from the data (markers) are compared with the predicted signal from the MC simulation, provided by Alpgen + Herwig and normalised to the NNLO prediction. In addition, physics backgrounds, also shown, have been added in proportion to the predictions from the MC simulation. The ratio between the expectation and the data is shown in the *lower plot*. The error bars shown on the data are statistical only

**Fig. 11:**
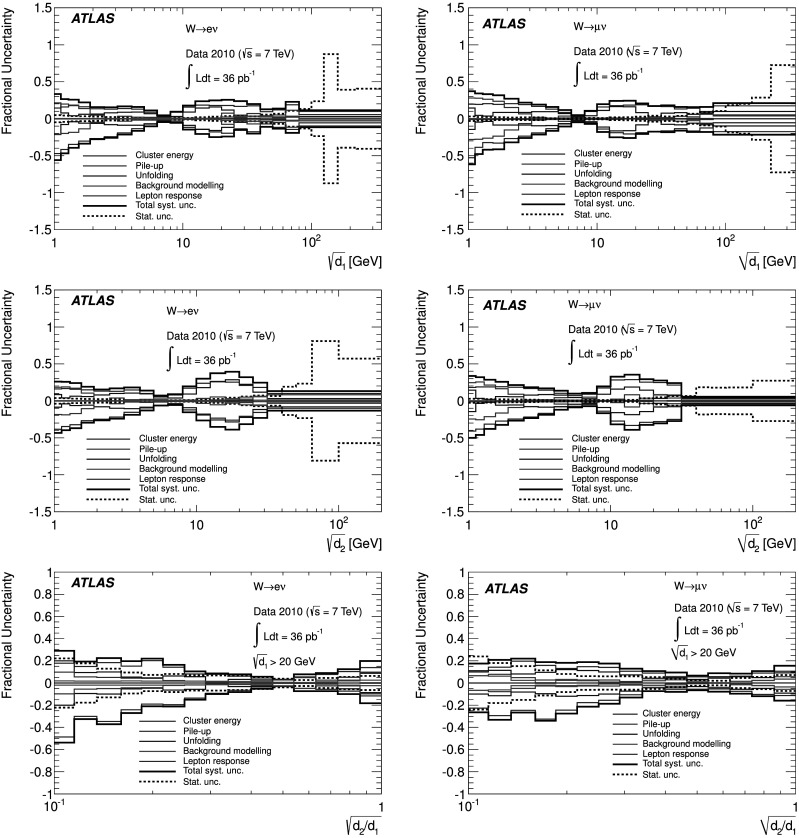
Summary of the systematic uncertainties on the measured particle-level distributions for $\sqrt{d_{1}}$ (*top*) and $\sqrt {d_{2}}$ (*middle*) and the ratio $\sqrt{d_{2}/d_{1}}$ (*bottom*) in the *W*→*eν* (*left*) and *W*→*μν* (*right*) channels

The distributions of the ratios $\sqrt{d_{k+1}/d_{k}}$ are displayed in Fig. 8. These probe the probability for a QCD emission of hardness $\sqrt{d_{k+1}}$ given a previous emission of scale $\sqrt{d_{k}}$. The Herwig parton shower used with both Alpgen and Mc@Nlo gives the best description of these observables. None of the ratio observables are expected to be dominated by perturbative effects, since the bulk of the events are collected near the lower threshold at $\sqrt{d_{k}}=20~\mathrm{GeV}$, and $\sqrt {d_{k+1}}$ is always softer than $\sqrt{d_{k}}$. The Powheg predictions, particularly for the case where Powheg is matched to Pythia6, deviate from the data in the ratio of the hardest and second-hardest clustering, $\sqrt{d_{1}/d_{0}}$. This is the only ratio observable that directly probes the NLO+PS matching in Powheg and Mc@Nlo.

## Conclusions

A first measurement of the *k*_T_ cluster splitting scales in *W* boson production at a hadron–hadron collider has been presented. The measurement was performed using the 2010 data sample from *pp* collisions at $\sqrt{s}=7~\mathrm{TeV}$ collected with the ATLAS detector at the LHC. The data correspond to approximately 36 pb^−1^ in both the electron and muon *W*-decay channels.

Results are presented for the four hardest splitting scales in a *k*_T_ cluster sequence, and ratios of these splitting scales. Backgrounds were subtracted and the results were corrected for detector effects to allow a comparison to different generator predictions at particle level. A weighted combination was performed to optimise the precision of the measurement. The dominant systematic uncertainties on the measurements originate from the cluster energy scale, pileup and the unfolding procedure.

The degree of agreement between various Monte Carlo simulations with the data varies strongly for different regions of the observables. The hard tails of the distributions are significantly better described by the multi-leg generators Alpgen + Herwig and Sherpa, which include exact tree-level matrix elements, than by the NLO+PS generators Mc@Nlo and Powheg. This also holds true for the hardest clustering, $\sqrt{d_{0}}$, even though it is formally predicted at the same QCD leading-order accuracy by all of these generators.

In the soft regions of the splitting scales, larger variations between all generators become evident. The generators based on the Herwig parton shower provide a good description of the data, while the Sherpa and Powheg+Pythia predictions do not reproduce the soft regions of the measurement well.

With this discriminating power the data thus test the resummation shape generated by parton showers and the extent to which the shower accuracy is preserved by the different merging and matching methods used in these Monte Carlo simulations.

## Footnotes

1 ATLAS uses a right-handed coordinate system with its origin at the nominal interaction point (IP) in the centre of the detector and the *z*-axis along the beam pipe. The *x*-axis points from the IP to the centre of the LHC ring, and the *y*-axis points upward. Cylindrical coordinates (*r*,*ϕ*) are used in the transverse plane, *ϕ* being the azimuthal angle around the beam pipe. The pseudorapidity is defined in terms of the angle *θ* as *η*=−lntan(*θ*/2).
